# Associations between pesticide mixtures applied near home during pregnancy and early childhood with adolescent behavioral and emotional problems in the CHAMACOS study

**DOI:** 10.1097/EE9.0000000000000150

**Published:** 2021-05-05

**Authors:** Carly Hyland, Patrick T. Bradshaw, Robert B. Gunier, Ana M. Mora, Katherine Kogut, Julianna Deardorff, Sharon K. Sagiv, Asa Bradman, Brenda Eskenazi

**Affiliations:** a Center for Environmental Research and Children’s Health (CERCH), School of Public Health, University of California at Berkeley, Berkeley, California; b Division of Epidemiology, School of Public Health, University of California, Berkeley, Berkeley, California; c Central American Institute for Studies on Toxic Substances (IRET), Universidad Nacional, Heredia, Costa Rica; d Department of Public Health, School of Social Sciences, Humanities, and Arts, University of California, Merced, Merced, California

**Keywords:** Pesticides, Glyphosate, Neonicotinoids, Organophosphates, Neurodevelopment, Bayesian methods, Children’s health, Adolescent health

## Abstract

Studies suggest that exposure to pesticides during pregnancy and early childhood is associated with adverse child neurodevelopment. Research to date has focused primarily on exposure to single pesticides or pesticide classes in isolation; there are little data on the effect of exposure to pesticide mixtures on child and adolescent neurodevelopment.

**Methods:**

Using California’s Pesticide Use Reporting database, we estimated agricultural pesticide use within 1 km of the home during the prenatal and postnatal (ages 0–5 years) periods among participants in the Center for the Health Assessment for Mothers and Children of Salinas (CHAMACOS) birth cohort. We implemented a Bayesian Hierarchical linear mixed-effects model to examine associations with maternal- and youth-reported behavioral and emotional problems from the Behavior Assessment System for Children, 2nd edition (BASC-2) at ages 16 and 18 years (n = 593).

**Results:**

We observed mostly null associations between pesticide applications and neurobehavioral outcomes. There were some trends of modestly increased internalizing behaviors and attention problems in association with organophosphate insecticide use near the home during the prenatal period. In the postnatal period, a two-fold increase in glyphosate applications was associated with more youth-reported depression (β = 1.2, 95% credible intervals [CrI] = 0.2, 2.2), maternal-reported internalizing behaviors (β = 1.23, 95% CrI = 0.2, 2.3), and anxiety (β = 1.2, 95% CrI = 0.2, 2.3). We observed some protective associations with imidacloprid during the prenatal period, particularly in sex-specific analyses.

**Conclusions:**

We found only some subtle associations between some pesticides and neurobehavioral outcomes. This study extends previous work by considering potential exposure to mixtures of pesticides.

## Abstract 2

Supplemental Digital Content is available in the text.

**What this study adds**

Prenatal and early-life exposure to organophosphate pesticides has been associated with adverse child neurodevelopment; however, data gaps exist regarding the impact of exposure to mixtures of pesticides. We employ Bayesian Hierarchical Models to examine associations of agricultural use of neurotoxic pesticides near the home during pregnancy and early childhood and adolescent neurobehavior in the Centers for the Health Assessment of Mothers and Children of Salinas cohort. This study extends previous work by considering potential exposure to mixtures of pesticides and is the first to examine associations of pesticides with behavior problems measured longitudinally into adolescence and young adulthood.

## Introduction

Evidence from longitudinal cohort studies indicates that biomarkers of pesticide exposure and residential proximity to agricultural pesticide applications during pregnancy and early childhood may be associated with adverse child neurodevelopment, including poorer cognition^1–6^ and increased hyperactivity/inattention^7–9^ and traits related to autism spectrum disorders.^10–13^ Despite relatively consistent findings for outcomes assessed during early and middle childhood, previous studies have only followed children up to the age of 12 years, and data gaps exist regarding the persistence of pesticide–neurodevelopment associations into adolescence and young adulthood.

Epidemiologic studies to date have focused primarily on exposure to single pesticides or pesticide classes at a time, which may result in biased measures of association due to copollutant confounding by other pesticides.^14^ In particular, previous research has examined the neurodevelopmental impacts of exposure to organophosphate (OP) pesticides in isolation. These studies have largely relied on urinary biomarkers such as dialkylphosphate (DAP) metabolites, which are nonspecific, to characterize exposure; less is known about the effects of specific OPs with varying levels of toxicity.^15^ Additionally, agricultural use of pesticides such as pyrethroids, neonicotinoids, and glyphosate has increased substantially in the United States and globally in recent decades^16–18^; yet, few longitudinal studies have examined their potential impacts on human health and neurobehavioral development.^5^

Bayesian methods have become increasingly utilized in epidemiologic analyses of chemical mixtures due to their ability to simultaneously model multiple highly correlated exposure variables.^14,19,20^ A particular advantage of Bayesian Hierarchical Modeling (BHM) is that it allows correlated exposures to “borrow” information from each other,^21^ resulting in more precise effect estimates.^19,20,22^ These estimators also reduce the potential for extreme exposure–outcome associations, addressing concerns regarding multiple comparisons,^19,23,24^ and produce highly interpretable results.

Because many pesticides lack biomarkers or are cost-prohibitive to analyze in biological samples, recent analyses have used geospatial methods to characterize potential exposure to a range of pesticides.^4,5,11,25^ In the Center for the Health Assessment of Mothers and Children of Salinas (CHAMACOS) cohort study, we are able to leverage California’s unique and comprehensive Pesticide Use Reporting (PUR) database to characterize agricultural pesticide applications near participants’ residences, allowing us to examine associations with potential exposure to mixtures of pesticides, including those that are now the most widely used in agriculture. In a previous analysis in our cohort, we found that participants living in the areas of highest cumulative pesticide use during the prenatal period had intelligence quotient deficits of approximately 7 points at the age of 7 years compared with those living in areas of the lowest pesticide use.^5^

Here, we investigate associations of agricultural applications of neurotoxic pesticides within 1 km of the home during pregnancy and early childhood with maternal- and self-reported behavioral and emotional problems at ages 16 and 18 years in the CHAMACOS cohort. This analysis extends previous research by employing BHM to examine associations with specific pesticides while accounting for correlated coexposures. This is the first study to examine associations of prenatal or early-life pesticide exposure with behavioral or emotional problems measured longitudinally into adolescence and young adulthood.

## Methods

### Study population

CHAMACOS is a longitudinal birth cohort study investigating the developmental impacts of environmental exposures among children born in the Salinas Valley, an agricultural region of Monterey County, California. The initial cohort (CHAM1) included pregnant women who met eligibility criteria (≥18 years old, <20-week gestation, Spanish- or English-speaking, qualified for low-income health insurance, and planning to deliver at the county hospital). CHAM1 participants were recruited in community clinics serving predominantly low-income Latino patients in 1999–2000. Of the 1,130 eligible women, 601 (53.2%) agreed to participate in the study. Of the 601 women enrolled at baseline, 527 (88%) remained in the study and delivered a live-born singleton and 337 (56%) remained in the study through the child’s 9-year assessment. In 2009–2011, we expanded the cohort and recruited an additional 305 9-year-old Salinas Valley residents whose mothers met eligibility criteria (≥18 years at delivery, Spanish- or English-speaking, qualified for low-income health insurance during pregnancy, delivered child in local hospital, and had sought prenatal care in the first trimester). CHAM2 participants were recruited via newspaper and radio announcements advertising a study on the health effects of pesticides and environmental chemicals at local elementary schools, churches, libraries, food banks, and community events. A total of 595 CHAMACOS participants (CHAM1 and CHAM2) remained in the cohort through the 16-year study visit. As of March 2020, when data collection was paused due to the COVID-19 pandemic, 478 CHAMACOS participants had also completed their 18-year study visits.

Mothers of CHAM1 participants were interviewed twice during pregnancy, after delivery, and throughout childhood. CHAM2 mothers completed a comprehensive baseline interview when their children were 9 years old. Mothers of CHAM1 and CHAM2 participants completed identical assessments when their children were 10.5, 12, 14, 16, and 18 years of age; CHAMACOS youth participants were interviewed directly starting at the age of 10.5 years. We restricted the current analyses to participants whose prenatal (n = 814) or early childhood (n = 443) residential history could be geocoded for pesticide exposure assessment and who had a maternal- or youth-reported neurobehavioral assessment from the 16-year (n = 594) or 18-year (n = 494) study visits. We excluded participants with medical conditions such as Down syndrome, autism, and hydrocephalus that could affect neurodevelopmental assessments (n = 6). The total sample size with data on the exposure and outcome for the 16-year analyses was 578 for prenatal and 428 for postnatal; the total for 18-year analyses was 476 for prenatal and 381 for postnatal.

The University of California Berkeley Committee for the Protection of Human Subjects approved all study activities, and we obtained written informed consent from all mothers at all study visits. We obtained youth written assent at the age of 16 years and written consent at the age of 18 years.

### Behavioral assessment

At the 16- and 18-year assessments, bilingual psychometricians administered the Behavior Assessment for Children, second edition (BASC-2)^26^ to mothers in their dominant language and the youth completed the BASC-2 Self-Report of Personality. We examined maternal- and youth-reported scores for four individual scales (hyperactivity, attention problems, depression, and anxiety) and the internalizing problems composite scale. In addition, we examined maternal-reported scores for the externalizing problems composite scale (there is no externalizing composite score for the youth-reported BASC-2 Self-Report of Personality). We examined BASC-2 age- and sex-standardized T-scores (M = 50, SD = 10).

### Estimation of agricultural pesticide use

To characterize potential exposure to a range of pesticides, including those for which biomarkers do not exist, we used California’s PUR database to characterize agricultural pesticide use near each participant’s residence during the prenatal and early childhood (0–5 years) periods, as has been described previously.^4,5^ We characterized agricultural applications of pesticides that (1) had evidence of neurotoxicity in humans or animals, (2) had more than 4,500 kg applied in Monterey County in the time period of interest, and (3) were used within 1 km of the home of at least 50% of CHAMACOS participants in the time period of interest (11 pesticides for the prenatal period and 12 pesticides for the postnatal period). We used the latitude and longitude coordinates from geocoded residential addresses, reported prospectively at all study visits for CHAM1 participants, and reported retrospectively at the 9- and 16-year visits from CHAM2 participants. We estimated the total amount of each pesticide that met these criteria applied within a 1-km radius of each residence. We selected a 1-km buffer because this distance has been used in previous epidemiologic analyses^4,5^ and has been shown to be most strongly correlated with concentrations of agricultural pesticides from house-dust samples.^27,28^ To account for the potential downwind transport of pesticides from the application site, we obtained data on wind direction from the closest meteorological station; these were located in Arroyo Seco, Castroville, King City, Salinas North, Salinas South, and Pajaro.^29^ We calculated wind frequency using the daily proportion of time the wind blew from each of eight directions during each time period (pregnancy and 0–5 years). We determined the direction of each Public Land Survey System centroid relative to residences and weighted pesticide use in a section according to the percentage of time that the wind blew from that direction for each time period. All pesticide use estimates were log_2_-tranformed and thus measures of association correspond to a two-fold increase in pesticide use.

### Covariates

At each study visit, bilingual study staff administered structured questionnaires to ascertain participant characteristics. The following confounders were selected a priori using a directed acyclic graph^30^: maternal age (continuous), years spent in the United States (categorical: ≤5 years, >5 years but not born in United States, born in United States), education (categorical: ≤6th grade, 7th–12th grade, completed high school), and marital status (dichotomous: not married/living as married vs. married/living as married) at the time of delivery. We also included the following predictors of the outcome a priori: maternal depression status at the 9-year assessment (categorical: yes vs. no) assessed using the Center for Epidemiologic Studies Depression Scale,^31^ child sex (dichotomous) and exact age at assessment (continuous), Home Observation Measurement of the Environment-Short Form^32^ z-score at the 10.5-year visit (continuous) to assess enrichment in the home, household income at the time of assessment (categorical: at or below poverty line vs. above poverty line), and language of interview assessment for maternal-reported outcomes (dichotomous: English vs. Spanish; all youth completed assessments in English).

### Statistical analysis

We implemented a two-stage BHM^19,33–39^ to examine exposure–outcome associations with all pesticides included simultaneously. In the first stage, we regressed each BASC outcome on the exposures and covariates in a single linear mixed-effects model with a random subject-specific effect as: (E[Y|X,W]) = α + Xβ + Wγ + u; where X is the vector of all pesticides, W is the vector of confounders, and u is a normally distributed subject-specific random effect. In the second stage, we modeled the exposure effects (β) as a function of an exchangeability matrix Z, coefficient vector π, and residual error δ (normally distributed with mean zero and variance τ^2^) as: β = Zπ + δ. We used a Z matrix with indicator variables (0/1) for the class to which each individual pesticide belongs, incorporating our a priori expectation that pesticides from the same class would exert similar effects of the outcome. For the primary analyses, we included only pesticide classes that had >1 pesticide in the Z matrix (i.e., OPs). We specified vague second-stage priors for individual pesticides not included in the Z matrix. For the postnatal analyses, we included a second Z matrix in which we adjusted for the 11 pesticides that were included in the prenatal analyses. The Bayesian framework allowed us to automatically account for missing outcomes for any participants missing data from a particular BASC domain, but who completed a neurobehavioral assessment at 16 and/or 18 years. We present β effect estimates and 95% credible intervals (CrIs) for each pesticide predicted from the first-stage model.

As suggested by the previous study,^40^ we specified vague priors on some model parameters (α, γ, and π) and prespecified the variance for δ (i.e., τ) based on background information. We selected a value of τ that assumed that β parameters would lie within ± 0.5 SD of the mean of the BASC outcome of interest in our population (i.e., from −5 to 5 in the normative sample). We specified models in a Fully Bayesian framework^21^ and estimated the posterior distribution of all model parameters via Markov Chain Monte Carlo sampling.^41^ We summarized the posterior distributions of these parameters by estimating the posterior median and 95% CrIs through Markov Chain Monte Carlo sampling^42^ using Just Another Gibbs Sampler.^43^ Models were run with 50,000 iterations after an initial burn-in of 10,000. Convergence was assessed graphically using trace plots, autocorrelation plots, and density plots,^41^ and statistically using the Geweke test^44^ and Gelman–Rubin test statistic.^45^ All analyses were conducted using R Studio Version 1.2.1335 (R Foundation for Statistical Computing, Vienna, Austria).

We conducted sex-specific analyses by including an interaction term between each pesticide and child sex in the first stage model. For sex-specific analyses, we included all pesticides in the second-stage model, as all pesticides would benefit from shrinkage due to the pesticide × sex interaction term (as compared with the primary analyses, where we only included pesticides with >1 pesticide in a class in the second-stage model, as described previously).

### Sensitivity analyses

We examined the robustness of our results by conducting sensitivity analyses in which we varied the specification of the Z matrix. First, we used a Z matrix in which we included indicator variables (0/1) for the class to which each individual pesticide belongs (i.e., OPs, carbamates, pyrethroids, neonicotinoids, fungicides, herbicides; Table S1; http://links.lww.com/EE/A135) for all pesticide classes, as opposed to excluding classes with only one pesticide from the Z matrix, as in the main analyses. In the second sensitivity analysis, we indicated whether each OP was a diethyl or dimethyl using a 0/1 indicator variable and incorporated the benchmark dose_10_, as used by the US Environmental Protection Agency for cumulative OP risk assessments^46^ (Table S2; http://links.lww.com/EE/A135). In addition to the hierarchical models, we also ran multivariable linear mixed-effects regression models in which we included all exposures simultaneously without specifying a second-stage model.

## Results

A total of 593 participants completed a maternal- or youth-reported BASC assessment at the 16- and/or 18-year study visits and provided residential history during pregnancy and/or childhood (ages 0–5 years). Most mothers were born in Mexico (87%) and nearly half had spent <5 years in the United States before delivery and had a sixth-grade education or less (Table 1). About 51% of the youth participants included in these analyses were girls.

**Table 1.** Sociodemographic characteristics of Center for the Health Assessment for Mothers and Children of Salinas study participants with 16- or 18-year neurobehavioral assessments and data on agricultural pesticide use near home during prenatal or postnatal (0–5 years) periods (n = 593).

Characteristic	n (%) or median (P25 to P75)
Maternal/household characteristics	
Age at enrollment (years)	26.0 (22.0 to 30.0)
Country of birth	
Mexico or other	519 (88.6)
United States	67 (11.4)
Years in the United States at delivery	
≤5 years	280 (47.7)
>5 years, but not born in United States	254 (43.3)
Born in United States	53 (9.0)
Education at baseline	
≤6th grade	258 (44.0)
7th–12th grade	194 (33.0)
≥High school graduate	135 (23.0)
Marital status at baseline	
Not married/living as married	106 (18.1)
Married/living as married	481 (81.9)
Maternal depression at 9-year visit (≥16 CES-D score)^a^	
No	417 (71.0)
Yes	170 (29.0)
Household income at 16-year assessment^a^	
At or below poverty level	333 (56.7)
Above poverty level	254 (43.3)
Language of 16-year maternal assessment^a^	
English	72 (12.5)
Spanish	506 (87.5)
HOME z-score at 10.5-year assessment^b^	0.2 (−0.6 to 0.6)
Child characteristics	
Child’s sex	
Boy	286 (48.7)
Girl	301 (51.3)
Exact age at 16-year assessment	16.3 (16.1 to 16.5)
Exact age at 18-year assessment	18.0 (18.0 to 18.1)

^a^Missing data filled in from data collected at earlier or later time points. n = 41 participants missing maternal depression at 9-year assessment; 7 missing poverty status at 16-year assessment; 16 missing maternal language of 16-year assessment.

^b^Missing data filled in from earlier or later assessments for 13 participants missing HOME score at 10.5-year assessment; filled in as median HOME score observed for population included in this analysis for one participant missing HOME score at all visits.

CES-D indicates Center for Epidemiologic Studies Depression Scale; HOME, Home Observation Measurement of the Environment.

The distributions of wind-adjusted neurotoxic pesticide applications within 1 km of the home during the prenatal and postnatal periods, as well as the total kilograms of pesticides applied in Monterey County in the years 2000 and 2005 (reflecting general trends in pesticide use during the prenatal and postnatal periods), are shown in Table 2. In general, applications of pesticides were highly correlated with each other during both the prenatal (Figure S1; http://links.lww.com/EE/A135) and postnatal (Figure S2; http://links.lww.com/EE/A135) periods. Individual OPs had some of the highest correlation coefficients (ρ = 0.4–0.9 during both prenatal and postnatal periods). Correlations coefficients for individual pesticides ranged from 0.50 to 0.71 across the prenatal and postnatal periods (Table 2).

**Table 2.** Total pesticide use in Monterey County in 2000 and 2005 and distributions of wind-adjusted agricultural pesticide applications within 1 km of maternal residence during prenatal and postnatal periods^a^

	Kilograms used (2000)	Kilograms used (2005)	Prenatal				Postnatal				Spearman correlation coefficient for prenatal and postnatal periods
			P25	P50	P75	Max	P25	P50	P75	Max	
Organophosphate insecticides											
Acephate	40,077	22,340	0.22	1.12	2.07	6.29	1.69	3.09	4.35	6.98	0.60
Chlorpyrifos	30,691	30,459	0.19	0.92	2.03	6.76	1.11	2.67	4.22	7.59	0.63
Diazinon	50,999	73,707	1.07	1.95	3.02	7.01	2.53	3.79	5.43	8.47	0.55
Malathion	30,490	29,513	0.00	0.31	1.47	6.68	0.92	2.14	3.89	7.86	0.31
Oxydemeton methyl	31,084	33,330	0.20	1.00	1.97	5.77	1.55	2.94	4.19	8.32	0.64
Naled^b^	13,090	7,839	0.00	0.00	0.54	3.93	0.00	0.98	2.48	6.37	0.50
Dimethoate	20,259	18,948	0.10	0.53	1.50	5.01	0.89	2.08	3.56	6.73	0.71
Carbamate insecticides											
Methomyl	35,371	28,843	0.22	0.82	1.86	4.91	1.50	2.71	3.77	7.33	0.51
Pyrethroid insecticides											
Permethrin	11,869	10,467	0.10	0.47	1.16	4.12	0.59	1.49	2.85	5.70	0.61
Neonicotinoid insecticides											
Imidacloprid	8,729	5,753	0.15	0.41	0.89	3.32	0.71	1.42	2.33	4.74	0.55
Fungicides											
Mn-fungicides	161,154	169,887	1.62	3.13	4.32	5.30	3.85	5.43	6.76	10.02	0.60
Herbicides											
Glyphosate	44,236	55,886	0.00	0.07	1.23	4.51	0.91	2.01	3.23	6.75	0.53

^a^Prenatal period accounts for 9 months of pregnancy and postnatal period accounts for child ages 0–5 years.

^b^Included in postnatal, but not in prenatal analysis.

### Associations with pesticide applications during prenatal period

We observed largely negligible associations between pesticide use near the home during pregnancy and neurobehavioral outcomes. There were some subtle associations of chlorpyrifos use and increased internalizing behaviors from both maternal- and youth-report. More specifically, each two-fold increase in chlorpyrifos applications was associated with increased BASC-2 T-scores for maternal-reported depression (β = 1.0, 95% CrI = −0.2, 2.1; Table 3) and youth-reported depression and internalizing problems (β = 1.1, 95% CrI = −0.1, 2.3; β = 1.0, 95% CrI = −0.2, 2.2, respectively; Table 4). For maternal depression only, we observed stronger associations among girls than among boys (boys: β = 0.7, 95% CrI = −0.7, 2.1; girls: β = 1.7, 95% CrI = 0.1, 3.3; Table S3; http://links.lww.com/EE/A135). We also observed some isolated associations of increased youth-reported attention problems in association with applications of the OPs diazinon and dimethoate during pregnancy (β = 1.2, 95% CrI = 0.0, 2.5 and β = 1.9, 95% CrI = 0.0, 3.6, respectively; Table 4).

**Table 3.** Adjusted^a^ associations [β (95% credible intervals)] of two-fold increase in pesticide use within 1 km of residence *during pregnancy* with *maternal*-*reported* behavioral and emotional problems at age 16 and 18 years using linear mixed-effects Bayesian Hierarchical Modeling (n = 1,049; *k* = 587)

	Internalizing problems	Depression	Anxiety	Externalizing problems	Hyperactivity	Attention problems
Organophosphates						
Acephate	0.1 (−1.4, 1.5)	−0.1 (−1.6, 1.3)	0.5 (−0.9, 2.9)	−0.5 (−1.5, 0.7)	−0.3 (−1.5, 0.8)	−0.7 (−2.1, 0.7)
Chlorpyrifos	0.7 (−0.5, 1.9)	1.0 (−0.2, 2.1)	0.5 (−0.6, 1.6)	0.3 (−0.6, 1.1)	0.3 (−0.6, 1.2)	−0.1 (−1.2, 0.9)
Diazinon	0.0 (−1.4, 1.4)	−0.2 (−1.6, 1.2)	0.4 (−0.9, 1.8)	0.2 (−0.8, 1.3)	0.1 (−1.0, 1.1)	0.3 (−1.0, 1.6)
Malathion	0.2 (−0.6, 1.0)	0.6 (−0.2, 1.4)	−0.4 (−1.2, 0.4)	−0.1 (−0.7, 0.5)	−0.1 (−0.8, 0.5)	0.1 (−0.6, 0.9)
Oxydemeton methyl	−0.1 (−2.2, 2.0)	0.3 (−1.8, 2.4)	−0.3 (−2.4, 1.8)	−0.1 (−1.6, 1.5)	−0.4 (−2.1, 1.3)	0.2 (−1.8, 2.2)
Dimethoate	0.5 (−1.4, 2.4)	0.4 (−1.5, 2.3)	0.0 (−1.9, 1.9)	0.4 (−1.0, 1.9)	0.8 (−0.8, 2.3)	1.2 (−0.6, 3.0)
Carbamates						
Methomyl	0.0 (−1.2, 1.2)	−0.2 (−1.3, 1.1)	0.1 (−1.0, 1.3)	0.4 (−0.5, 1.3)	0.3 (−0.7, 1.2)	0.0 (−1.1, 1.1)
Pyrethroid						
Permethrin	−1.5 (−3.9, 0.9)	−1.3 (−3.8, 1.1)	−1.4 (−3.8, 0.9)	0.2 (−1.6, 2.1)	0.1 (−1.8, 2.0)	0.5 (−1.8, 2.7)
Neonicotinoid						
Imidacloprid	−0.2 (−3.3, 2.8)	0.0 (−3.1, 3.1)	−1.1 (−4.2, 1.8)	−0.3 (−2.5, 2.0)	−0.6 (−3.1, 1.8)	−2.4 (−5.3, 0.4)
Fungicide						
Mn-fungicides	0.0 (−1.3, 1.2)	−0.1 (−1.3, 1.1)	0.1 (−1.1, 1.3)	−0.3 (−1.2, 0.6)	0.0 (−1.0, 0.9)	0.1 (−1.1, 1.2)
Herbicide						
Glyphosate	0.2 (−0.5, 0.9)	−0.1 (−0.9, 0.6)	0.4 (−0.4, 1.1)	0.3 (−0.2, 0.9)	0.4 (−0.2, 1.0)	0.4 (−0.3, 1.1)

Notes: *k*, number of participants with data for at least one time point; n, number of observations from both time points. Higher score for each Behavior Assessment System for Children outcome indicates more symptomatic behavior.

^a^Models adjusted for maternal age at delivery, years in the United States, education at baseline, marital status at baseline, language of assessment, depression at 9-year assessment; child sex, child age at time of assessment, poverty status at time of assessment, Home Observation Measurement of the Environment score at 10.5-year assessment.

**Table 4.** Adjusted^a^ associations [β (95% credible intervals)] of two-fold increase in pesticide use within 1 km of residence *during pregnancy* with *youth-reported* behavioral and emotional problems at age 16 and 18 years using linear mixed-effects Bayesian Hierarchical Modeling (n = 1,032; *k* = 584)

	Internalizing problems	Depression	Anxiety	Hyperactivity	Attention problems
Organophosphates					
Acephate	0.0 (−1.5, 1.6)	0.2 (−1.3, 1.7)	−0.6 (−2.2, 1.0)	−0.1 (−1.5, 1.2)	0.2 (−1.2, 1.6)
Chlorpyrifos	1.0 (−0.2, 2.2)	1.1 (−0.1, 2.3)	0.6 (−0.7, 1.9)	−0.1 (−1.2, 1.0)	0.0 (−1.1, 1.1)
Diazinon	0.5 (−1,0, 1.9)	0.4 (−1.0, 1.8)	0.4 (−1.1, 1.9)	0.1 (−1.1, 1.3)	1.2 (0.0, 2.5)
Malathion	0.6 (−0.2, 1.4)	0.6 (−0.3, 1.4)	0.6 (−0.3, 1.5)	−0.1 (−0.8, 0.7)	0.0 (−0.7, 0.8)
Oxydemeton methyl	−1.1 (−3.3, 1.1)	−0.8 (−3.0, 1.4)	−0.5 (−2.9, 1.8)	−0.6 (−2.6, 1.3)	−1.4 (−3.3, 0.6)
Dimethoate	−0.6 (−2.6, 1.4)	−1.2 (−3.1, 0.8)	−0.4 (−2.5, 1.8)	0.6 (−1.2, 2.4)	1.9 (0.0, 3.6)
Carbamates					
Methomyl	0.0 (−1.3, 1.2)	−0.6 (−1.8, 0.6)	−0.1 (−1.5, 1.2)	0.3 (−0.8, 1.4)	−0.3 (−1.4, 0.8)
Pyrethroid					
Permethrin	−0.6 (−3.2, 1.9)	−0.9 (−3.3, 1.7)	−1.3 (−4.0, 1.4)	−0.7 (−3.0, 1.5)	−1.9 (−4.2, 0.3)
Neonicotinoid					
Imidacloprid	−0.4 (−3.6, 2.7)	0.2 (−2.9, 3.3)	−0.8 (−4.1, 2.5)	0.4 (−2.3, 3.2)	−1.6 (−4.4, 1.3)
Fungicide					
Mn-fungicides	0.1 (−1.2, 1.4)	0.3 (−1.0, 1.6)	0.6 (−0.8, 2.0)	−0.1 (−1.2, 1.0)	0.1 (−1.0, 1.3)
Herbicide					
Glyphosate	0.3 (−0.5, 1.0)	0.4 (−0.4, 1.1)	0.4 (−0.4, 1.2)	0.4 (−0.3, 1.1)	0.2 (−0.5, 0.8)

Notes: *k*, number of participants with data for at least one time point; n, number of observations from both time points. Higher score for each Behavior Assessment System for Children outcome indicates more symptomatic behavior.

^a^Models adjusted for maternal age at delivery, years in the United States, education at baseline, marital status at baseline, depression at 9-year assessment; child sex, child age at time of assessment, poverty status at time of assessment, Home Observation Measurement of the Environment score at 10.5-year assessment.

In contrast, we found associations of use of the neonicotinoid imidacloprid with fewer maternal- and youth-reported behavioral and emotional problems (Tables 3 and 4), particularly for girls (Table S3; http://links.lww.com/EE/A135 and Table S4; http://links.lww.com/EE/A135). Among all participants, each two-fold increase in imidacloprid use during the prenatal period was associated with decreased maternal-reported attention problems (β = −2.4, 95% CrI = −5.3, 0.4; Table 3), with evidence of stronger inverse associations among girls (boys: β = −2.2, 95% CrI = −4.8, 0.5; girls: β = −4.5, 95% CrI = −7.9, −0.9; Table S3; http://links.lww.com/EE/A135). Imidacloprid use was also associated with decreased maternal-reported internalizing problems and depression among girls, as well as youth-reported internalizing problems and anxiety among girls. We also observed that the use of the pyrethroid permethrin was associated with fewer youth-reported attention problems among all participants (β = −1.9, 95% CrI = −4.2, 0.3; Table 4). Table S5 (http://links.lww.com/EE/A135) summarizes associations observed in the prenatal period.

### Associations with pesticide applications during the postnatal period

The most consistent associations we observed for pesticide use during the postnatal period were for glyphosate and maternal- and youth-reported internalizing behaviors. Each two-fold increase in glyphosate applications was associated with increased maternal report of internalizing problems and anxiety (β = 1.3, 95% CrI = 0.2, 2.3 and β = 1.2, 95% CrI = 0.2, 2.3, respectively; Table 5) and youth report of depression (β = 1.2, 95% CrI = 0.2, 2.2; Table 6), with trends of stronger associations among girls (Table S6; http://links.lww.com/EE/A135 and Table S7; http://links.lww.com/EE/A135). Notably, in contrast, chlorpyrifos and naled were each associated with decreased maternal-reported anxiety (β = −1.7, 95% CrI = −3.3, −0.1 and β = −1.2, 95% CrI = −2.5, 0.0, respectively; Table 5), with stronger inverse associations for chlorpyrifos and maternal-reported anxiety among girls (boys: β = −0.9, 95% CrI = −2.7, 0.9; girls: β = −2.2, 95% CrI = −4.1, −0.3; Table S5; http://links.lww.com/EE/A135).

**Table 5.** Adjusted^a^ associations [β (95% credible intervals)] of two-fold increase in pesticide use within 1 km of residence *during childhood (0-5 years*) with *maternal-reported* behavioral and emotional problems at age 16 and 18 years using linear mixed-effects Bayesian Hierarchical Modeling (n = 797; *k* = 427)

	Internalizing problems	Depression	Anxiety	Externalizing problems	Hyperactivity	Attention problems
Organophosphates						
Acephate	0.8 (−1.0, 2.6)	0.5 (−1.3, 2.3)	1.0 (−0.9, 2.8)	0.8 (−0.6, 2.1)	1.6 (0.1, 3.0)	0.5 (−1.1, 2.1)
Chlorpyrifos	−1.0 (−2.5, 0.6)	−0.1 (−1.7, 1.6)	−1.7 (−3.3, −0.1)	0.1 (−1.1, 1.3)	0.1 (−1.2, 1.3)	0.9 (−0.6, 2.3)
Diazinon	1.1 (−0.9, 3.1)	1.3 (−0.8, 3.3)	0.1 (−1.9, 2.2)	0.0 (−1.5, 1.5)	0.2 (−1.3, 1.8)	1.2 (−0.7, 3.0)
Malathion	0.2 (−0.7, 1.3)	0.1 (−0.9, 1.1)	0.7 (−0.3, 1.7)	−0.4 (−1.1, 0.4)	−0.5 (−1.3, 0.3)	−0.9 (−1.8, 0.0)
Oxydemeton methyl	0.3 (−2.5, 3.0)	−0.4 (−3.1, 2.3)	0.5 (−2.2, 3.1)	−1.0 (−3.0, 1.0)	−2.4 (−4.5, −0.2)	−2.3 (−4.6, 0.2)
Naled	−0.7 (−2.0, 0.6)	−0.2 (−1.4, 1.1)	−1.2 (−2.5, 0.0)	0.4 (−0.5, 1.4)	0.3 (−0.7, 1.3)	0.8 (−0.4, 1.9)
Dimethoate	0.9 (−1.2, 3.1)	−0.3 (−2.4, 1.9)	1.0 (−1.2, 3.2)	0.7 (−1.0, 2.4)	0.8 (−0.9, 2.5)	−1.1 (−3.1, 0.9)
Carbamates						
Methomyl	0.1 (−1.6, 1.7)	0.6 (−1.1, 2.2)	−0.6 (−2.2, 1.0)	−0.2 (−1.5, 1.0)	−0.5 (−1.8, 0.8)	−0.9 (−2.4, 0.6)
Pyrethroid						
Permethrin	0.2 (−2.1, 2.6)	−0.2 (−2.6, 2.1)	0.5 (−1.8, 2.9)	0.0 (−1.8, 1.7)	−0.8 (−2.6, 1.1)	−0.8 (−2.9, 1.3)
Neonicotinoid						
Imidacloprid	−0.1 (−2.9, 2.7)	−0.5 (−3.3, 2.2)	1.0 (−1.7, 3.7)	0.1 (−2.0, 2.2)	1.2 (−1.0, 3.3)	1.5 (−0.9, 4.0)
Fungicide						
Mn-fungicides	−1.8 (−3.9, 0.4)	−0.9 (−3.1, 1.3)	−1.0 (−3.2, 1.1)	−0.3 (−2.0, 1.3)	0.2 (−1.5, 1.9)	0.9 (−1.1, 2.8)
Herbicide						
Glyphosate	1.3 (0.2, 2.3)	0.6 (−0.5, 1.6)	1.2 (0.2, 2.3)	0.1 (−0.7, 0.9)	0.1 (−0.8, 0.9)	0.0 (−0.9, 1.0)

Notes: *k*, number of participants with data for at least one time point; n, number of observations from both time points. Higher score for each Behavior Assessment System for Children outcome indicates more symptomatic behavior.

^a^Models adjusted for maternal age at delivery, years in the United States, education at baseline, marital status at baseline, language of assessment, depression at 9-year assessment; child sex, child age at time of assessment, poverty status at time of assessment, Home Observation Measurement of the Environment score at 10.5-year assessment, agricultural applications of 11 pesticides included in prenatal assessment during the prenatal period.

**Table 6.** Adjusted^a^ associations [β (95% credible intervals)] of two-fold increase in pesticide use within 1 km of residence *during childhood (0–5 years*) with *youth-reported* behavioral and emotional problems at age 16 and 18 years using linear mixed-effects Bayesian Hierarchical Modeling (n = 786; *k* = 426)

	Internalizing problems	Depression	Anxiety	Hyperactivity	Attention problems
Organophosphates					
Acephate	0.3 (−1.6, 2.1)	0.1 (−1.7, 1.8)	−0.8 (−2.8, 1.2)	0.8 (−0.9, 2.4)	−0.2 (−1.9, 1.5)
Chlorpyrifos	−1.1 (−2.7, 0.5)	−1.4 (−2.9, 0.2)	−0.9 (−2.6, 0.8)	0.1 (−1.4, 1.5)	0.1 (−1.4, 1.6)
Diazinon	0.3 (−1.7, 2.3)	0.4 (−1.5, 2.3)	0.7 (−1.5, 2.8)	0.3 (−1.6, 2.1)	−0.2 (−2.0, 1.7)
Malathion	−0.7 (−1.7, 0.3)	−0.4 (−1.3, 0.6)	−0.4 (−1.5, 0.7)	−0.3 (−1.3, 0.6)	−0.9 (−1.8, 0.1)
Oxydemeton methyl	−0.1 (−2.8, 2.7)	0.5 (−2.1, 3.1)	0.7 (−2.3, 3.5)	−1.8 (−4.3, 0.6)	−1.1 (−3.6, 1.4)
Naled	0.5 (−0.8, 1.7)	0.3 (−0.9, 1.5)	−0.3 (−1.7, 1.1)	0.0 (−1.1, 1.2)	1.2 (0.1, 2.4)
Dimethoate	2.0 (−0.2, 4.2)	1.5 (−0.6, 3.6)	1.5 (−0.8, 3.9)	2.0 (0.0, 3.9)	1.2 (−0.8, 3.2)
Carbamates					
Methomyl	−1.4 (−3.0, 0.2)	−1.1 (−2.6, 0.5)	−0.3 (−2.1, 1.5)	−0.8 (−2.3, 0.7)	−0.9 (−2.4, 0.6)
Pyrethroid					
Permethrin	0.3 (−2.0, 2.6)	0.0 (−2.2, 2.3)	0.5 (−2.0, 3.0)	−0.1 (−2.2, 2.1)	0.1 (−2.1, 2.3)
Neonicotinoid					
Imidacloprid	−0.7 (−3.4, 2.0)	−1.3 (−3.9, 1.2)	−1.5 (−4.4, 1.5)	−0.5 (−3.0, 2.0)	0.4 (−2.1, 3.0)
Fungicide					
Mn-fungicides	0.1 (−2.0, 2.3)	0.1 (−2.0, 2.2)	0.3 (−2.0, 2.6)	0.8 (−1.1, 2.8)	0.7 (−1.3, 2.7)
Herbicide					
Glyphosate	0.9 (−0.1, 2.0)	1.2 (0.2, 2.2)	0.9 (−0.3, 2.0)	−0.5 (−1.5, 0.4)	0.0 (−1.0, 1.0)

Notes: *k*, number of participants with data for at least one time point; *n*, number of observations from both time points. Higher score for each Behavior Assessment System for Children outcome indicates more symptomatic behavior.

^a^Models adjusted for maternal age at delivery, years in the United States, education at baseline, marital status at baseline, depression at 9-year assessment; child sex, child age at time of assessment, poverty status at time of assessment, Home Observation Measurement of the Environment score at 10.5-year assessment, agricultural applications of 11 pesticides included in prenatal assessment during the prenatal period.

We observed some associations of OP use postnatally and increased externalizing problems, though there were no consistent trends. For example, dimethoate was associated with increased youth-reported hyperactivity (β = 2.0, 95% CrI = 0.0, 3.9) and naled was associated with increased youth-reported attention problems (β = 1.2, 95% CrI = 0.1, 2.4). Additionally, acephate was associated with increased maternal-reported hyperactivity (β = 1.6, 95% CrI = 0.1, 3.0). We also observed some inverse associations, with the OPs oxydemeton methyl and malathion associated with decreased maternal- and youth-reported attention problems (β = −2.3, 95% CrI = −4.6, 0.2 and β = −0.9, 95% CrI = −1.8, 0.1, respectively). Table S8 (http://links.lww.com/EE/A135) summarizes associations observed in the prenatal period.

### Sensitivity analyses

Results from our sensitivity analyses were robust to variations of the specification of the Z matrix, and our overall interpretations were qualitatively the same (data not shown). Results were also very similar from multivariable models in which we included all exposure variables simultaneously without specifying the second-stage model (Table S9; http://links.lww.com/EE/A135 and Table S10; http://links.lww.com/EE/A135 for prenatal analyses and Table S11; http://links.lww.com/EE/A135-S12; http://links.lww.com/EE/A135 for postnatal analyses). Confidence intervals were slightly wider for some pesticides in multivariable linear mixed-effects regression models; however, our overall interpretation of the results was consistent with findings from the hierarchical analyses.

## Discussion

We observed mostly null associations of agricultural pesticide use near the home during critical periods of brain development and behavioral and emotional problems at ages 16 and 18 years among participants living in an intensive agricultural region. We observed some associations of use of the OP chlorpyrifos near the home during pregnancy and use of glyphosate near the home during early childhood with increased internalizing problems; however effect estimates were small. We also observed trends of fewer maternal- and youth-reported internalizing behaviors and attention problems in association with imidacloprid use near the home during pregnancy. This is the first study to examine longitudinal associations of agricultural pesticide use near the home during pregnancy or early childhood with behavioral problems during adolescence or young adulthood, a critical time for the manifestation of these outcomes.^47^ Our study also extends the previous research by investigating potential exposure to multiple classes of pesticides.

Previous studies examining associations of prenatal or postnatal OP exposure and child neurodevelopment have largely assessed exposure using nonspecific DAP metabolites, limiting inferences regarding associations with specific OP pesticides. In this analysis, we found associations of agricultural use of chlorpyrifos, a diethyl OP, during pregnancy with increased report of internalizing problems, depression, and anxiety from both mothers and youth; however, effect estimates were quite small. In the longitudinal Mount Sinai Children’s Environmental Health Center study, investigators found that higher prenatal dimethyl OP concentrations were associated with more BASC parent-reported internalizing problems among 141 children from ages 4 to 9 years.^48^ In addition to studies of children, chronic occupational OP exposure and history of acute OP poisonings have been associated with increased self-reported depression among farmworkers,^49–54^ and the Agricultural Health Study has observed some of the strongest associations for pesticides such as malathion, diazinon, and chlorpyrifos.^53^

We observed some isolated associations of increased youth-reported attention problems in association with applications of the OPs diazinon and dimethoate during the prenatal period. In previous analyses in this cohort, prenatal DAPs were associated with higher maternal-reported attention problems and psychometrician-assessed Attention Deficit Hyperactivity Disorder (ADHD) at the age of 5, but not at the age of 3.5 years.^7^ Additionally, in a longitudinal analysis of inner-city mothers and children in New York City, investigators found that prenatal chlorpyrifos concentrations were associated with increased attention and hyperactivity problems at 3 years.^8^ Notably, we did not observe associations of chlorpyrifos use during the prenatal or postnatal period with maternal- or youth-reported attention problems or hyperactivity in this analysis. Cross-sectional and case–control studies have also found associations between childhood OP exposure and more behavioral and attention problems^55^ and higher odds of having an ADHD diagnosis.^56,57^

We observed largely null associations of permethrin use near the home during either pregnancy or early childhood with maternal- or youth-reported behavioral or emotional problems. This is in contrast with previous studies showing associations of prenatal pyrethroid exposure with child behavior problems. Specifically, longitudinal studies in New York City and France have identified associations of prenatal biomarkers of pyrethroid exposure and more parent-reported behavioral and emotional problems, including internalizing problems, depression, and externalizing problems, among children ages 4–9 years.^58,59^ Results of cross-sectional studies investigating childhood pyrethroid exposure and behavioral outcomes have been more inconsistent. While one analysis of 1999–2002 data from the National Health and Nutrition Examination Survey found no association of pyrethroid exposure and parental report of ADHD among children ages 6–15 years,^60^ another analysis of National Health and Nutrition Examination Survey participants ages 8–15 years from 2001 to 2002 found that higher urinary levels of a nonspecific pyrethroid biomarker, 3-phenoxybenzoic acid, were associated with higher odds of an ADHD diagnosis and more hyperactive-impulsive symptoms.^61^ In the cross-sectional Canadian Health Measures Survey, two other pyrethroid biomarkers were associated with increased odds of parent-reported global total difficulties assessed using the Strengths and Difficulties Questionnaire among 779 children ages 6–11 years.^62^ It is possible that inconsistencies in findings from our study and previous analyses may be due to different exposure assessment methods or the age at which the outcome was assessed. Notably, each of these previous studies assessed exposure using urinary biomarkers, which are a more integrated measure of total pyrethroid exposure than PUR data. Residential pesticide use is one of the biggest risk factors for pyrethroid exposure,^63^ which would not be captured with our exposure assessment method.

We also observed associations of applications of the neonicotinoid imidacloprid during the prenatal period with fewer maternal- and youth-reported internalizing behaviors and attention problems, particularly among girls. While neonicotinoids are intended to be highly selective to insects^64^ and are thought to have low mammalian toxicity due to a lower affinity for binding to the nicotine acetylcholine receptor,^65,66^ few epidemiologic studies have examined their impacts on human health and significant data gaps exist.^66,67^ Toxicological studies suggest that gestational imidacloprid exposure may be associated with sensorimotor deficits in the offspring,^68^ and case studies indicate that acute neonicotinoid poisoning can result in adverse respiratory, cardiovascular, and neurologic outcomes.^66^ However, no studies to date have examined associations of prenatal or early-life neonicotinoid exposure with adolescent neurobehavior, using either frequentist or Bayesian mixtures models. It is possible that we observed null or protective effects for imidacloprid because our exposure assessment method—agricultural pesticide use—did not adequately capture imidacloprid exposure due to the physical properties and mode of application of neonicotinoids.^17,66,69,70^ Neonicotinoids are commonly applied as seed treatments,^71,72^ and more integrated exposure assessment methods, such as urinary biomarkers, may be needed to accurately characterize exposure. Neonicotinoids are now the most widely used class of insecticides worldwide and use continues to rise.^17,73^ Additional studies, potentially using biomarkers of exposure, are needed to examine the neurodevelopmental impacts of neonicotinoids.

We observed relatively consistent associations of glyphosate use near the home during the postnatal period across maternal- and youth-reported internalizing problems, depression, and anxiety. Very few epidemiologic studies have examined neurodevelopmental outcomes associated with glyphosate exposure, though toxicology studies have shown neurotoxic effects such as depressive behavior^74,75^ and poorer locomotor activity^75–78^ and recognition memory.^76,79^ In one previous case–control study using PUR data, investigators found that glyphosate use within 2 km of the mother’s residence during pregnancy was associated with increased odds of autism spectrum disorder.^13^ Additionally, case studies of acute poisoning have also suggested that glyphosate may have direct impacts on neurotoxicity and Parkinsonism after chronic exposures.^80–82^ Glyphosate is the most widely used pesticide in the United States and worldwide, with global use increasing about 15-fold since the introduction of genetically engineered glyphosate-tolerant crops in 1996.^18^ Agricultural and nonagricultural use of glyphosate has continued to skyrocket since the exposure periods of interest for the present analysis, and additional studies are needed to investigate whether early exposure to current levels of glyphosate use may be associated with child or adolescent neurodevelopment.

We did not observe consistent trends over prenatal and postnatal analyses. Previous examinations of urinary biomarkers of OP pesticides and neurodevelopment in CHAMACOS and other studies have observed stronger effects for exposures occurring prenatally.^1,7,83,84^ Notably, the exposure period of interest was 9 months for pregnancy and 5 years for childhood exposures, and thus prenatal and postnatal effect estimates are not directly comparable in the present analysis.

We did observe some consistencies for associations with specific pesticides across maternal and youth report. For example, effect estimates for internalizing behaviors in association with chlorpyrifos use during pregnancy and glyphosate use during early childhood were similar across maternal and youth report. Previous studies have reported relatively poor agreement between maternal and youth report of adolescent psychopathology,^85^ particularly for internalizing behaviors.^86–88^ Mothers may be more reliable reporters of adolescent externalizing behaviors, which may be more easily observed by others, as opposed to depression or anxiety, which the participant may choose not to disclose to caregivers.^86^

Although it is difficult to elucidate potential mechanisms of actions of specific pesticides from epidemiology studies in which humans are exposed to a mixture of pesticides, evidence from animal studies suggests that possible mechanisms may include changes in levels of neurotransmitters,^75^ inhibition of axonal growth,^89,90^ alteration of voltage-gated sodium channel function,^91–93^ increased oxidative stress,^94–96^ and damage to or decreased synthesis of brain DNA.^97–100^ The inhibition of acetylcholinesterase was long proposed as one of the primary neurodevelopmental mechanisms of action of OP and carbamate pesticides; however, there is growing evidence from human and animal studies that these pesticides may exert deleterious impacts on neurodevelopment at levels of exposure below which acetylcholinesterase inhibition would occur.^83,101^ For example, OPs may disrupt neurotransmitter systems including norepinephrine, dopamine, and serotonin,^102–107^ which could influence emotional and behavioral problems such as aggression, depression, and ADHD that have been associated with OP exposure in previous epidemiologic studies.^7,56,108^ Toxicology studies have shown that developmental glyphosate exposure may also impact cholinergic and glutamatergic neurotransmission, increase oxidative stress, and induce neural cell death in the hippocampus.^74^ There is consistent evidence from epidemiologic and animal studies that fetuses and young children, who are undergoing periods of rapid brain and nervous system development,^109^ are particularly susceptible to the potential neurotoxic effects of pesticides^109,110^ and may experience neurobehavioral abnormalities at doses that would not be toxic to adults.^111–113^

Our study has several strengths and limitations. One of the biggest limitations is that applications of pesticides near the home are not a direct measure of exposure and reliance on PUR data may result in measurement error. Previous analyses suggest that PUR data are correlated with environmental concentrations of OPs, but not pyrethroids, in homes,^27,114^ and data gaps exist regarding how well reliance on PUR may capture exposure to other pesticides such as neonicotinoids or glyphosate. The precision of the exposure assessment was likely independent of the outcomes of interest and would thus result in nondifferential misclassification that may have contributed to our mostly null findings. We were also only able to characterize potential exposure to pesticides based on use near the maternal residence, and not in other areas the mothers and children may have spent time during the prenatal and postnatal periods, such as work and childcare. Additionally, while CHAM1 participants reported their residential address at all study visits, addresses and timing of household moves were reported retrospectively for CHAM2 participants and may be prone to error.

Strengths include a well-characterized cohort with rich collection of data, including longitudinal neurobehavioral measures from two reporters (i.e., mothers and youth). While it has been well established that prenatal and, to a lesser extent, postnatal OP pesticide exposure is associated with adverse child neurodevelopment, a number of data gaps exist. Previous studies have examined associations among children followed up to age 12 years, and ours is the first to examine the persistence of pesticide–neurodevelopment associations into adolescence and young adulthood. Moreover, previous investigations have largely examined single pesticides or pesticide classes in isolation, which may result in bias from copollutant confounding.^14^ Many studies have also relied on DAPs or other nonspecific biomarkers that reflect only very recent exposures,^115^ resulting in data gaps regarding the impact of specific pesticides with varying degrees of toxicity. By leveraging California’s unique and comprehensive PUR database, we were able to examine associations with multiple neurotoxic pesticides, including those that lack biomarkers. We employed BHM as a principled approach to examine associations with all pesticides included in a single model, allowing for estimation of mutually adjusted exposure effects that are more stable and interpretable than with other approaches to multiple exposure modeling (e.g., simultaneous inclusion of all exposure variables).^19,20,22^ While multiple methods are being developed to examine environmental mixtures, BHM has many advantages in that it allows the incorporation of a priori information; facilitates a “borrowing” of information across similar exposures^21^ that results in estimates with lower mean squared error and interval estimate coverage closer to the nominal level; reduces the potential for extreme exposure–outcome associations, addressing concerns regarding multiple comparisons^19,23,24^; and produces highly interpretable results.

## Conclusion

This is the first study to examine associations of applications of mixtures of neurotoxic pesticides near the home during pregnancy or early childhood, critical periods of brain development, and neurobehavioral outcomes assessed during adolescence or young adulthood. Adolescence is an important time for the manifestation of these behavioral outcomes^47^ and may have important downstream effects on other outcomes, including impaired school performance, juvenile delinquency, increased risk-taking behavior, substance abuse, adult crime, and future psychopathology.^116–118^ We found mostly null or modest associations between pesticides and neurobehavioral outcomes. Pesticide use trends have shifted drastically since the prenatal and postnatal exposure periods for children in this study; as many OPs are being phased out from residential and agricultural use due to evidence of neurotoxicity to the developing brain, it is increasingly important to study the safety of their replacements.

## ACKNOWLEDGMENTS

We gratefully acknowledge the CHAMACOS laboratory and field staff, students, community partners, and the participants and their families.

## Supplementary Material


