# Supplementary material for: Associations between pesticide mixtures applied near home during pregnancy and early childhood with adolescent behavioral and emotional problems in the CHAMACOS study
Source: Environ Epidemiol. 2021 May 5;5(3):e150. doi: 10.1097/EE9.0000000000000150 (PMC8196094; doi:10.1097/EE9.0000000000000150)
Supplement: Supplementary file 1 [file ee9-5-e150-s001.docx]

| **Table S1**. Exchangeability matrix for Bayesian Hierarchical Model (BHM) for prenatal analyses from Sensitivity Analysis 1^a^. | | | | | | | | | | | |
| --- | --- | --- | --- | --- | --- | --- | --- | --- | --- | --- | --- |
|  | OP |  | Carbamate |  | Pyrethroid |  | Neonicotinoid |  | Fungicide |  | Herbicide |
| Acephate | 1 |  | 0 |  | 0 |  | 0 |  | 0 |  | 0 |
| Chlorpyrifos | 1 |  | 0 |  | 0 |  | 0 |  | 0 |  | 0 |
| Diazinon | 1 |  | 0 |  | 0 |  | 0 |  | 0 |  | 0 |
| Malathion | 1 |  | 0 |  | 0 |  | 0 |  | 0 |  | 0 |
| Oxydemeton methyl | 1 |  | 0 |  | 0 |  | 0 |  | 0 |  | 0 |
| Dimethoate | 1 |  | 0 |  | 0 |  | 0 |  | 0 |  | 0 |
| Methomyl | 0 |  | 1 |  | 0 |  | 0 |  | 0 |  | 0 |
| Permethrin | 0 |  | 0 |  | 1 |  | 0 |  | 0 |  | 0 |
| Imidacloprid | 0 |  | 0 |  | 0 |  | 1 |  | 0 |  | 0 |
| Maneb & Mancozeb | 0 |  | 0 |  | 0 |  | 0 |  | 1 |  | 0 |
| Glyphosate | 0 |  | 0 |  | 0 |  | 0 |  | 0 |  | 1 |
| ^a^Sensitivity analysis in which all pesticide classes were included in the Z matrix. | | | | | | | | | | | |

| **Table S2**. Exchangeability matrix for Bayesian Hierarchical Model (BHM) for prenatal analyses, Sensitivity Analysis 2^a^. | | | | | | |
| --- | --- | --- | --- | --- | --- | --- |
|  |  | DE |  | DM |  | BMD_10_ |
| Acephate |  | 0 |  | 1 |  | 0.99 |
| Chlorpyrifos |  | 1 |  | 0 |  | 1.48 |
| Diazinon |  | 1 |  | 0 |  | 6.24 |
| Malathion |  | 0 |  | 1 |  | 313.91 |
| Oxydemeton methyl |  | 0 |  | 1 |  | 0.09 |
| Dimethoate |  | 0 |  | 1 |  | 0.25 |
| ^b^Sensitivity analysis in which only OP pesticides were included in the Z matrix (with designation of whether OP was a diethyl or dimethyl pesticide and benchmark dose) and null priors implemented for remaining pesticides. | | | | | | |

| **Table S3.** Adjusted associations [β (95% CrI)] of two-fold increase in pesticide use within 1 km of residence *during pregnancy* with *maternal-reported* behavioral and emotional problems at age 16 and 18 years using linear mixed effects Bayesian Hierarchical Modeling (BHM), stratified by sex (boys: *n*=506, *k*=286; girls: *n*=543, *k*=301). | | | | | | | | | | | | | | | | | | |
| --- | --- | --- | --- | --- | --- | --- | --- | --- | --- | --- | --- | --- | --- | --- | --- | --- | --- | --- |
|  |  | Internalizing problems | |  | Depression | |  | Anxiety | |  | Externalizing problems | |  | Hyperactivity | |  | Attention problems | |
|  |  | Boys | Girls |  | Boys | Girls |  | Boys | Girls |  | Boys | Girls |  | Boys | Girls |  | Boys | Girls |
| OPs |  |  |  |  |  |  |  |  |  |  |  |  |  |  |  |  |  |  |
| Acephate |  | -0.3 (-2.0, 1.3) | -1.0 (-3.1, 1.1) |  | -0.2 (-1.9, 1.4) | -0.9 (-3.1, 1.3) |  | 0.4 (-1.1, 2.0) | -0.3 (-2.4, 1.8) |  | -0.1 (-1.3, 1.0) | -0.3 (-1.8, 1.3) |  | 0.1 (-1.2, 1.3) | -0.2 (-1.8, 1.4) |  | -0.9 (-2.3, 0.6) | 0.3 (-1.5, 2.3) |
| Chlorpyrifos |  | 0.6 (-0.7, 2.0) | 0.8 (-0.8, 2.5) |  | 0.7 (-0.7, 2.1) | 1.7 (0.1, 3.3) |  | 0.1 (-1.2, 1.5) | 1.1 (-0.5, 2.6) |  | 0.3 (-0.7, 1.3) | 0.9 (-0.2, 2.0) |  | 0.1 (-0.9, 1.1) | 0.9 (-0.2, 2.1) |  | -0.6 (-1.8, 0.6) | 0.6 (-0.7, 2.0) |
| Diazinon |  | 0.3 (-1.3, 1.9) | -0.2 (-2.1, 1.8) |  | -0.5 (-2.1, 1.2) | -0.9 (-2.8, 1.2) |  | 0.3 (-1.3, 1.9) | -0.2 (-2.1, 1.8) |  | 0.3 (-0.9, 1.4) | -0.5 (-2.0, 0.9) |  | 0 (-1.2, 1.1) | -0.5 (-2.0, 0.9) |  | 0.8 (-0.7, 2.1) | -0.1 (-1.9, 1.6) |
| Malathion |  | 0.6 (-0.5, 1.6) | 0.0 (-1.1, 1.2) |  | 0.9 (-0.1, 2) | 0.7 (-0.5, 1.8) |  | 0.1 (-0.9, 1.2) | -0.7 (-1.8, 0.5) |  | -0.3 (-1.1, 0.4) | 0.5 (-0.3, 1.4) |  | -0.2 (-0.9, 0.6) | 0.0 (-0.8, 0.9) |  | 0.1 (-0.8, 1.0) | 0.4 (-0.5, 1.4) |
| Oxydemeton methyl |  | -0.6 (-2.9, 1.7) | 0.1 (-3.0, 3.2) |  | -0.2 (-2.6, 2.1) | 0.8 (-2.4, 4.0) |  | -0.7 (-3.1, 1.6) | 0.5 (-2.6, 3.5) |  | -0.3 (-2.0, 1.3) | 0.0 (-2.2, 2.3) |  | -0.6 (-2.4, 1.1) | -0.5 (-2.9, 1.8) |  | -0.6 (-2.6, 1.5) | -0.4 (-3.1, 2.4) |
| Dimethoate |  | 0.8 (-1.4, 3.0) | 0.2 (-2.4, 2.7) |  | 1.4 (-0.8, 3.6) | -1.1 (-3.8, 1.5) |  | 0.1 (-2.0, 2.3) | -1 (-3.5, 1.5) |  | 1.4 (-0.2, 3.0) | -1.5 (-3.4, 0.4) |  | 1.9 (0.3, 3.6) | -0.9 (-2.9, 1.0) |  | 2.0 (0.1, 4.0) | 1.1 (-1.2, 3.4) |
| Carbamates |  |  |  |  |  |  |  |  |  |  |  |  |  |  |  |  |  |  |
| Methomyl |  | -0.8 (-2.2, 0.6) | 0.9 (-0.7, 2.6) |  | -1.4 (-2.8, 0.0) | 1.4 (-0.3, 3.0) |  | -0.4 (-1.8, 1.0) | 0.1 (-1.5, 1.8) |  | -0.2 (-1.2, 0.9) | 1.9 (0.7, 3.1) |  | -0.2 (-1.3, 0.8) | 1.5 (0.2, 2.7) |  | 0.0 (-1.3, 1.3) | 0.3 (-1.2, 1.7) |
| Pyrethroid |  |  |  |  |  |  |  |  |  |  |  |  |  |  |  |  |  |  |
| Permethrin |  | -0.9 (-3.3, 1.6) | 0.7 (-2.3, 3.7) |  | -0.3 (-2.9, 2.2) | 1.0 (-2.0, 4.1) |  | -1.3 (-3.8, 1.1) | 1.2 (-1.8, 4.2) |  | 0.3 (-1.5, 2.1) | 2.6 (0.5, 4.8) |  | -0.4 (-2.4, 1.4) | 1.7 (-0.6, 4.0) |  | 0.1 (-2.1, 2.4) | 1.0 (-1.8, 3.7) |
| Neonicotinoid |  |  |  |  |  |  |  |  |  |  |  |  |  |  |  |  |  |  |
| Imidacloprid |  | 0.3 (-2.7, 3.3) | -4.7 (-8.6, -0.8) |  | 0.7 (-2.2, 3.8) | -4.4 (-8.3, -0.4) |  | -0.2 (-3.2, 2.7) | -3.0 (-6.8, 0.9) |  | -0.5 (-2.6, 1.7) | -2.8 (-5.7, 0.0) |  | -0.1 (-2.4, 2.2) | -2.6 (-5.6, 0.4) |  | -2.2 (-4.8, 0.5) | -4.5 (-7.9, -0.9) |
| Fungicide |  |  |  |  |  |  |  |  |  |  |  |  |  |  |  |  |  |  |
| Mn-Fungicides |  | 0.4 (-1.1, 1.9) | 0.5 (-1.1, 2.0) |  | 0.4 (-1.1, 1.9) | 0.3 (-1.3, 1.9) |  | 0.6 (-0.9, 2.1) | 0.3 (-1.3, 1.8) |  | -0.5 (-1.6, 0.6) | -0.6 (-1.8, 0.5) |  | -0.3 (-1.4, 0.8) | 0.2 (-1, 1.4) |  | -0.1 (-1.4, 1.3) | -0.2 (-1.6, 1.1) |
| Herbicide |  |  |  |  |  |  |  |  |  |  |  |  |  |  |  |  |  |  |
| Glyphosate |  | -0.5 (-1.5, 0.4) | 0.0 (-1.0, 1.1) |  | -1.2 (-2.2, -0.3) | -0.1 (-1.1, 0.9) |  | 0.3 (-0.6, 1.2) | -0.1 (-1.1, 0.9) |  | 0.0 (-0.7, 0.6) | 0.3 (-0.4, 1.0) |  | 0.0 (-0.7, 0.7) | 0.5 (-0.3, 1.2) |  | -0.1 (-0.9, 0.7) | 0.5 (-0.4, 1.4) |
| Notes: *k*, number of participants with data for at least one time point; *n*, number of observations from both time points. Higher score for each BASC outcome indicates more symptomatic behavior.  ^a^Models adjusted for maternal age at delivery, years in the U.S., education at baseline, marital status at baseline, language of assessment, depression at 9Y assessment; child age at time of assessment, poverty status at time of assessment, HOME score at 10.5Y assessment, agricultural applications of 11 pesticides included in prenatal assessment during the prenatal period. | | | | | | | | | | | | | | | | | | |

| **Table S4.** Adjusted associations [β (95% CrI)] of two-fold increase in pesticide use within 1 km of residence *during pregnancy* with *youth-reported* behavioral and emotional problems at age 16 and 18 years using linear mixed effects Bayesian Hierarchical Modeling (BHM), stratified by sex (boys: *n*=495, *k*=285; girls: *n*=537, *k*=299). | | | | | | | | | | | | | | | |
| --- | --- | --- | --- | --- | --- | --- | --- | --- | --- | --- | --- | --- | --- | --- | --- |
|  |  | Internalizing problems | |  | Depression | |  | Anxiety | |  | Hyperactivity | |  | Attention problems | |
|  |  | Boys | Girls |  | Boys | Girls |  | Boys | Girls |  | Boys | Girls |  | Boys | Girls |
| OPs |  |  |  |  |  |  |  |  |  |  |  |  |  |  |  |
| Acephate |  | 0.3 (-1.4, 2.1) | 0.4 (-1.7, 2.7) |  | 0.1 (-1.5, 1.8) | -0.4 (-2.4, 1.7) |  | 0.7 (-1.1, 2.5) | -0.6 (-2.9, 1.7) |  | 0.4 (-1.1, 1.9) | -0.8 (-2.7, 1.1) |  | 0.2 (-1.3, 1.7) | -0.4 (-2.4, 1.5) |
| Chlorpyrifos |  | 1.3 (-0.1, 2.7) | 1.2 (-0.3, 2.7) |  | 1.0 (-0.3, 2.3) | 1.4 (-0.2, 2.9) |  | 0.0 (-1.4, 1.5) | 0.9 (-0.7, 2.6) |  | -0.3 (-1.5, 0.9) | -0.2 (-1.6, 1.2) |  | 0.3 (-1.0, 1.5) | 0.2 (-1.2, 1.6) |
| Diazinon |  | 0.1 (-1.5, 1.7) | 1.4 (-0.6, 3.4) |  | 0 (-1.5, 1.6) | 1 (-0.9, 3) |  | -0.4 (-2.1, 1.3) | 1.1 (-1.0, 3.1) |  | 0.0 (-1.4, 1.4) | 0.8 (-1.0, 2.6) |  | 1.0 (-0.5, 2.4) | 1.4 (-0.5, 3.2) |
| Malathion |  | 0.9 (-0.2, 2.0) | -0.3 (-1.5, 0.8) |  | 0.7 (-0.3, 1.7) | -0.3 (-1.4, 0.8) |  | 0.9 (-0.2, 2.1) | -0.2 (-1.5, 0.9) |  | -0.9 (-1.8, 0.1) | 0.5 (-0.5, 1.5) |  | 0.4 (-0.6, 1.3) | -0.7 (-1.7, 0.3) |
| Oxydemeton methyl |  | -1.1 (-3.5, 1.3) | -1.6 (-4.7, 1.5) |  | -0.9 (-3.2, 1.5) | -0.3 (-3.3, 2.7) |  | -0.9 (-3.5, 1.5) | -0.7 (-4, 2.6) |  | 0.3 (-1.9, 2.4) | -0.2 (-2.9, 2.6) |  | -1.1 (-3.2, 1.1) | -1.5 (-4.4, 1.3) |
| Dimethoate |  | -0.9 (-3.1, 1.3) | -1.3 (-4.0, 1.3) |  | -0.8 (-2.9, 1.4) | -3 (-5.5, -0.4) |  | -0.6 (-3, 1.7) | 0.5 (-2.3, 3.4) |  | -0.2 (-2.1, 1.9) | 0.5 (-1.7, 2.9) |  | 1.5 (-0.5, 3.6) | 1.0 (-1.4, 3.2) |
| Carbamates |  |  |  |  |  |  |  |  |  |  |  |  |  |  |  |
| Methomyl |  | -0.4 (-1.9, 1.1) | 1.2 (-0.5, 3.0) |  | -0.8 (-2.3, 0.6) | 0.9 (-0.7, 2.5) |  | -0.7 (-2.2, 0.9) | 0.0 (-1.8, 1.8) |  | 0.4 (-0.8, 1.8) | 0.6 (-0.9, 2.1) |  | 0.0 (-1.3, 1.4) | 0.2 (-1.3, 1.7) |
| Pyrethroid |  |  |  |  |  |  |  |  |  |  |  |  |  |  |  |
| Permethrin |  | -0.4 (-2.9, 2.2) | -1.0 (-4.1, 2.2) |  | -0.3 (-2.8, 2.2) | 0.0 (-3, 3) |  | -0.6 (-3.4, 2.1) | -1.9 (-5.3, 1.3) |  | -0.4 (-2.7, 1.9) | -0.7 (-3.5, 2.0) |  | -1.8 (-4.0, 0.6) | -1.0 (-3.8, 1.9) |
| Neonicotinoid |  |  |  |  |  |  |  |  |  |  |  |  |  |  |  |
| Imidacloprid |  | -0.8 (-4.0, 2.3) | -3.9 (-7.9, 0.0) |  | -0.1 (-3.0, 2.8) | -2.0 (-5.9, 1.9) |  | -0.5 (-3.7, 2.7) | -4.3 (-8.4, 0.0) |  | 1.9 (-0.8, 4.6) | -1.2 (-4.7, 2.3) |  | -1.5 (-4.3, 1.1) | -2.1 (-5.5, 1.4) |
| Fungicide |  |  |  |  |  |  |  |  |  |  |  |  |  |  |  |
| Mn-Fungicides |  | 0.7 (-0.9, 2.2) | 0.1 (-1.4, 1.5) |  | 0.8 (-0.7, 2.3) | 0.2 (-1.3, 1.7) |  | 1.4 (-0.2, 3.0) | 0.8 (-0.9, 2.5) |  | -0.6 (-2.0, 0.7) | -0.4 (-1.8, 1.0) |  | 0.3 (-1.0, 1.7) | 0.3 (-1.1, 1.8) |
| Herbicide |  |  |  |  |  |  |  |  |  |  |  |  |  |  |  |
| Glyphosate |  | -0.2 (-1.1, 0.8) | 0.5 (-0.5, 1.5) |  | 0.3 (-0.6, 1.2) | 0.1 (-0.8, 1.1) |  | 0.0 (-1.0, 1.0) | 0.6 (-0.5, 1.7) |  | 0.3 (-0.5, 1.1) | 0.5 (-0.4, 1.4) |  | -0.3 (-1.1, 0.5) | 0.3 (-0.7, 1.2) |
| Notes: *k*, number of participants with data for at least one time point; *n*, number of observations from both time points. Higher score for each BASC outcome indicates more symptomatic behavior.  ^a^Models adjusted for maternal age at delivery, years in the U.S., education at baseline, marital status at baseline, depression at 9Y assessment; child age at time of assessment, poverty status at time of assessment, HOME score at 10.5Y assessment, agricultural applications of 11 pesticides included in prenatal assessment during the prenatal period. | | | | | | | | | | | | | | | |

| **Table S5.** Summary of associations of two-fold increase in pesticide use within 1 km of residence *during pregnancy* with maternal- and youth-reported behavioral and emotional problems at age 16 and 18 years using linear mixed effects Bayesian Hierarchical Modeling (BHM). | | | | | | | | | | | | |
| --- | --- | --- | --- | --- | --- | --- | --- | --- | --- | --- | --- | --- |
|  | Internalizing problems | | Depression | | Anxiety | | Externalizing problems | | Hyperactivity | | Attention problems | |
|  | Maternal | Youth | Maternal | Youth | Maternal | Youth | Maternal | Youth | Maternal | Youth | Maternal | Youth |
| Organophosphates |  |  |  |  |  |  |  |  |  |  |  |  |
| Acephate |  |  |  |  |  |  |  |  |  |  |  |  |
| Chlorpyrifos |  | **+** | **+** | **+** |  |  |  |  |  |  |  | **+** |
| Diazinon |  |  |  |  |  |  |  |  |  |  |  |  |
| Malathion |  |  |  |  |  |  |  |  |  |  |  |  |
| Oxydemeton methyl |  |  |  |  |  |  |  |  |  |  |  | **+** |
| Dimethoate |  |  |  |  |  |  |  |  |  |  |  |  |
| Carbamates |  |  |  |  |  |  |  |  |  |  |  |  |
| Methomyl |  |  |  |  |  |  |  |  |  |  |  |  |
| Pyrethroid |  |  |  |  |  |  |  |  |  |  |  | **-** |
| Permethrin |  |  |  |  |  |  |  |  |  |  |  |  |
| Neonicotinoid |  |  |  |  |  |  |  |  |  |  |  |  |
| Imidacloprid |  |  |  |  |  |  |  |  |  |  | - |  |
| Fungicide |  |  |  |  |  |  |  |  |  |  |  |  |
| Mn-Fungicides |  |  |  |  |  |  |  |  |  |  |  |  |
| Herbicide |  |  |  |  |  |  |  |  |  |  |  |  |
| Glyphosate |  |  |  |  |  |  |  |  |  |  |  |  |
| Notes: “+” indicates more symptomatic behavior; “-“ indicates less symptomatic behavior; blank cell indicates null association | | | | | | | | | | | | |

| **Table S6.** Adjusted associations [β (95% CrI)] of two-fold increase in pesticide use within 1 km of residence *during childhood (0-5 years)* with *maternal-reported* behavioral and emotional problems at age 16 and 18 years using linear mixed effects Bayesian Hierarchical Modeling (BHM), stratified by sex (boys: *n*=379, *k*=204; girls: *n*=418, k=223). | | | | | | | | | | | | | | | | | | |  |
| --- | --- | --- | --- | --- | --- | --- | --- | --- | --- | --- | --- | --- | --- | --- | --- | --- | --- | --- | --- |
|  |  | Internalizing problems | |  | Depression | |  | Anxiety | |  | Externalizing problems | |  | Hyperactivity | |  | Attention problems | | |
|  |  | Boys | Girls |  | Boys | Girls |  | Boys | Girls |  | Boys | Girls |  | Boys | Girls |  | Boys | Girls | |
| OPs |  |  |  |  |  |  |  |  |  |  |  |  |  |  |  |  |  |  | |
| Acephate |  | -0.5 (-2.4, 1.4) | 1.7 (-0.6, 3.9) |  | -0.4 (-2.2, 1.5) | 1.5 (-0.7, 3.7) |  | 0.0 (-1.9, 1.8) | 1.2 (-1.1, 3.4) |  | 0.6 (-0.8, 2.0) | 1.6 (0.0, 3.3) |  | 1.0 (-0.5, 2.6) | 1.9 (0.1, 3.8) |  | 0.4 (-1.3, 2.2) | -0.1 (-2.2, 2.0) | |
| Chlorpyrifos |  | 0.0 (-1.8, 1.8) | -1.5 (-3.4, 0.3) |  | 0.6 (-1.2, 2.3) | -0.8 (-2.7, 1.0) |  | -0.9 (-2.7, 0.9) | -2.2 (-4.1, -0.3) |  | 0.5 (-0.9, 1.8) | -0.6 (-2.0, 0.8) |  | 0.3 (-1.2, 1.7) | -0.6 (-2.1, 0.8) |  | 0.2 (-1.5, 1.8) | 1.0 (-0.8, 2.7) | |
| Diazinon |  | 1.4 (-0.5, 3.2) | 0.2 (-2.5, 2.8) |  | 1.4 (-0.5, 3.3) | 0.9 (-1.7, 3.6) |  | 0.9 (-0.9, 2.8) | -1.1 (-3.7, 1.6) |  | 0.1 (-1.3, 1.5) | -0.4 (-2.4, 1.6) |  | 0.8 (-0.6, 2.3) | -0.2 (-2.3, 1.9) |  | 2.1 (0.4, 3.9) | 0.7 (-1.8, 3.1) | |
| Malathion |  | 0.4 (-0.8, 1.5) | 0.0 (-1.1, 1.1) |  | 0.7 (-0.4, 1.8) | 0.0 (-1.1, 1.1) |  | 0.6 (-0.5, 1.7) | 0.1 (-1.0, 1.2) |  | -0.1 (-1.0, 0.7) | -0.2 (-1.0, 0.6) |  | -0.2 (-1.1, 0.7) | -0.2 (-1.1, 0.6) |  | -0.1 (-1.1, 1.0) | -0.6 (-1.7, 0.4) | |
| Oxydemeton methyl |  | -0.5 (-3.1, 2.1) | 1.4 (-1.8, 4.5) |  | -0.9 (-3.6, 1.8) | 0.1 (-3.0, 3.2) |  | -0.1 (-2.7, 2.6) | 1.2 (-2.0, 4.4) |  | -1.6 (-3.6, 0.3) | 0.2 (-2.2, 2.5) |  | -2.8 (-5.0, -0.7) | -0.6 (-3.1, 1.9) |  | -2.0 (-4.5, 0.5) | -0.3 (-3.2, 2.6) | |
| Naled |  | -0.6 (-2.1, 0.8) | -0.7 (-2.1, 0.8) |  | -0.6 (-2.0, 0.9) | 0.3 (-1.1, 1.7) |  | -1.3 (-2.7, 0.2) | -0.8 (-2.3, 0.6) |  | 0.1 (-1.0, 1.2) | 0.3 (-0.8, 1.4) |  | 0.2 (-1.0, 1.3) | 0.3 (-0.9, 1.5) |  | 0.0 (-1.4, 1.3) | 1.3 (-0.1, 2.7) | |
| Dimethoate |  | 1.1 (-1.2, 3.5) | 1.1 (-1.4, 3.7) |  | 0.2 (-2.1, 2.5) | 0.3 (-2.3, 2.8) |  | 1.0 (-1.3, 3.3) | 0.9 (-1.7, 3.5) |  | 0.2 (-1.4, 2.0) | 1.2 (-0.7, 3.1) |  | 0.8 (-1.0, 2.6) | 0.9 (-1.3, 2.9) |  | -1.5 (-3.6, 0.6) | -1.1 (-3.5, 1.3) | |
| Carbamates |  |  |  |  |  |  |  |  |  |  |  |  |  |  |  |  |  |  | |
| Methomyl |  | 0.4 (-1.3, 2.1) | -0.5 (-2.6, 1.7) |  | 1.2 (-0.5, 2.8) | -0.8 (-2.9, 1.3) |  | -0.5 (-2.3, 1.1) | -0.8 (-2.9, 1.3) |  | 0.6 (-0.7, 1.9) | -2.0 (-3.5, -0.4) |  | 0.5 (-0.9, 1.8) | -1.5 (-3.2, 0.2) |  | 0.6 (-0.9, 2.2) | -3.0 (-5.0, -1.0) | |
| Pyrethroid |  |  |  |  |  |  |  |  |  |  |  |  |  |  |  |  |  |  | |
| Permethrin |  | -0.7 (-2.8, 1.4) | 2.9 (-0.2, 5.9) |  | -0.8 (-2.9, 1.3) | 1.1 (-1.9, 4.2) |  | -0.8 (-2.9, 1.3) | 2.8 (-0.2, 5.9) |  | 0.0 (-1.5, 1.6) | -0.3 (-2.6, 1.9) |  | -1.0 (-2.7, 0.7) | -1.9 (-4.4, 0.5) |  | -0.6 (-2.6, 1.4) | -2.5 (-5.3, 0.3) | |
| Neonicotinoid |  |  |  |  |  |  |  |  |  |  |  |  |  |  |  |  |  |  | |
| Imidacloprid |  | 1.0 (-1.5, 3.5) | -0.4 (-3.8, 3.0) |  | 0.4 (-2.1, 2.9) | -0.5 (-3.9, 2.9) |  | 2.1 (-0.5, 4.6) | 1.6 (-1.8, 5.0) |  | 0.4 (-1.5, 2.3) | 1.8 (-0.8, 4.3) |  | 1.2 (-0.8, 3.2) | 3.2 (0.5, 5.9) |  | 1.0 (-1.4, 3.4) | 3.9 (0.7, 7.1) | |
| Fungicide |  |  |  |  |  |  |  |  |  |  |  |  |  |  |  |  |  |  | |
| Mn-Fungicides |  | -1.4 (-3.5, 0.7) | -2.8 (-5.3, -0.3) |  | -1.2 (-3.3, 0.8) | -1.6 (-4.1, 0.8) |  | -0.6 (-2.7, 1.5) | -1.5 (-4.0, 1.1) |  | -0.6 (-2.1, 1.0) | -0.9 (-2.8, 0.9) |  | -0.4 (-2.1, 1.2) | -0.6 (-2.5, 1.3) |  | -0.2 (-2.1, 1.8) | 0.8 (-1.4, 3.0) | |
| Herbicide |  |  |  |  |  |  |  |  |  |  |  |  |  |  |  |  |  |  | |
| Glyphosate |  | 0.2 (-0.9, 1.4) | 1.9 (0.7, 3.2) |  | 0.0 (-1.1, 1.2) | 1.0 (-0.2, 2.2) |  | 0.4 (-0.7, 1.6) | 1.6 (0.3, 2.8) |  | -0.2 (-1.0, 0.7) | 0.9 (-0.1, 1.8) |  | -0.3 (-1.2, 0.6) | 0.8 (-0.2, 1.8) |  | -0.4 (-1.4, 0.7) | 0.4 (-0.8, 1.6) | |
| Notes: *k*, number of participants with data for at least one time point; *n*, number of observations from both time points. Higher score for each BASC outcome indicates more symptomatic behavior.  ^a^Models adjusted for maternal age at delivery, years in the U.S., education at baseline, marital status at baseline, language of assessment, depression at 9Y assessment; child age at time of assessment, poverty status at time of assessment, HOME score at 10.5Y assessment, agricultural applications of 11 pesticides included in prenatal assessment during the prenatal period. | | | | | | | | | | | | | | | | | | |  |

| **Table S7.** Adjusted associations [β (95% CrI)] of two-fold increase in pesticide use within 1 km of residence *during childhood (0-5 years)* with *youth-reported* behavioral and emotional problems at age 16 and 18 years^a^ using linear mixed effects Bayesian Hierarchical Modeling (BHM), stratified by sex (boys: *n*=370, *k*=203; girls: *n*=416, k=223). | | | | | | | | | | | | | | | |
| --- | --- | --- | --- | --- | --- | --- | --- | --- | --- | --- | --- | --- | --- | --- | --- |
|  |  | Internalizing problems | |  | Depression | |  | Anxiety | |  | Hyperactivity | |  | Attention problems | |
|  |  | Boys | Girls |  | Boys | Girls |  | Boys | Girls |  | Boys | Girls |  | Boys | Girls |
| OPs |  |  |  |  |  |  |  |  |  |  |  |  |  |  |  |
| Acephate |  | 0.3 (-1.6, 2.2) | 0.3 (-2.1, 2.7) |  | 0.3 (-1.6, 2.2) | 0.6 (-1.8, 2.8) |  | -1.1 (-3.2, 1.0) | -0.8 (-3.2, 1.7) |  | 1.4 (-0.4, 3.2) | 1.4 (-0.7, 3.5) |  | 0.2 (-1.6, 2.1) | -0.9 (-3.1, 1.3) |
| Chlorpyrifos |  | -0.2 (-2.1, 1.7) | -1.2 (-3.1, 0.7) |  | 0.2 (-1.5, 2.0) | -1.5 (-3.3, 0.3) |  | -0.9 (-2.9, 1.1) | -0.7 (-2.7, 1.3) |  | 0.3 (-1.3, 2.0) | -0.6 (-2.3, 1.1) |  | 0.8 (-0.9, 2.4) | -0.5 (-2.2, 1.2) |
| Diazinon |  | -0.4 (-2.4, 1.6) | 1.8 (-1.0, 4.5) |  | -0.4 (-2.3, 1.4) | 1.8 (-0.8, 4.5) |  | 0.0 (-2.0, 2.1) | 1.4 (-1.5, 4.3) |  | 0.0 (-1.8, 1.7) | 0.2 (-2.3, 2.6) |  | -0.3 (-2.1, 1.5) | 0.3 (-2.2, 2.8) |
| Malathion |  | -0.4 (-1.6, 0.7) | -0.3 (-1.4, 0.9) |  | -0.2 (-1.4, 0.9) | -0.5 (-1.6, 0.6) |  | -0.1 (-1.4, 1.2) | -0.3 (-1.5, 0.9) |  | -0.3 (-1.4, 0.7) | 0.1 (-0.9, 1.1) |  | -0.5 (-1.5, 0.6) | -1.1 (-2.1, 0.0) |
| Oxydemeton methyl |  | -0.9 (-3.6, 1.8) | -0.8 (-4.1, 2.4) |  | 0.0 (-2.6, 2.7) | -0.3 (-3.4, 2.9) |  | 0.2 (-2.7, 3.1) | 0.1 (-3.3, 3.4) |  | -2.5 (-4.9, -0.1) | -2.5 (-5.4, 0.3) |  | -1.6 (-4.1, 0.9) | -2.0 (-5.0, 0.9) |
| Naled |  | 0.1 (-1.4, 1.6) | -0.2 (-1.7, 1.3) |  | -0.7 (-2.1, 0.7) | 0.9 (-0.5, 2.4) |  | -0.3 (-1.9, 1.3) | -0.3 (-1.9, 1.2) |  | -0.2 (-1.5, 1.2) | 0.3 (-1.0, 1.7) |  | 0.2 (-1.2, 1.6) | 2.0 (0.6, 3.4) |
| Dimethoate |  | 1.2 (-1.2, 3.6) | 3.0 (0.3, 5.7) |  | 0.0 (-2.2, 2.3) | 1.8 (-0.7, 4.2) |  | 1.0 (-1.5, 3.5) | 2.2 (-0.6, 5.0) |  | 1.8 (-0.3, 3.9) | 2.6 (0.2, 4.9) |  | 0.4 (-1.8, 2.5) | 3.2 (0.8, 5.7) |
| Carbamates |  |  |  |  |  |  |  |  |  |  |  |  |  |  |  |
| Methomyl |  | -0.5 (-2.3, 1.3) | -1.4 (-3.5, 0.7) |  | 0.0 (-1.7, 1.7) | -1.4 (-3.4, 0.6) |  | 0.8 (-1.0, 2.7) | -0.9 (-3.1, 1.4) |  | -1.2 (-2.8, 0.3) | -1.0 (-2.9, 0.9) |  | -0.6 (-2.3, 1.0) | -2.0 (-3.9, -0.1) |
| Pyrethroid |  |  |  |  |  |  |  |  |  |  |  |  |  |  |  |
| Permethrin |  | 0.2 (-2.1, 2.3) | 0.4 (-2.8, 3.5) |  | -0.3 (-2.4, 1.8) | 0.8 (-2.2, 3.9) |  | 0.2 (-2.1, 2.5) | 0.7 (-2.6, 3.9) |  | -0.4 (-2.3, 1.6) | -0.3 (-3.0, 2.5) |  | -0.6 (-2.6, 1.4) | 0.2 (-2.7, 3.2) |
| Neonicotinoid |  |  |  |  |  |  |  |  |  |  |  |  |  |  |  |
| Imidacloprid |  | -0.2 (-2.7, 2.5) | -3.3 (-6.8, 0.3) |  | 0.5 (-1.9, 3.0) | -3.2 (-6.6, 0.1) |  | -0.6 (-3.3, 2.2) | -3.7 (-7.3, 0.0) |  | 1.5 (-0.8, 3.8) | -1.1 (-4.3, 2.0) |  | 1.4 (-1.0, 3.8) | 0.3 (-2.9, 3.5) |
| Fungicide |  |  |  |  |  |  |  |  |  |  |  |  |  |  |  |
| Mn-Fungicides |  | 0.7 (-1.5, 2.8) | 0.1 (-2.5, 2.6) |  | 0.0 (-2.1, 2.1) | -0.1 (-2.4, 2.3) |  | 0.9 (-1.3, 3.2) | 0.7 (-1.9, 3.4) |  | 1.2 (-0.7, 3.1) | 0.9 (-1.3, 3.2) |  | 1.3 (-0.7, 3.2) | 1.1 (-1.2, 3.4) |
| Herbicide |  |  |  |  |  |  |  |  |  |  |  |  |  |  |  |
| Glyphosate |  | 0.2 (-1.0, 1.4) | 1.8 (0.5, 3.1) |  | 0.7 (-0.4, 1.8) | 1.6 (0.4, 2.8) |  | 0.5 (-0.8, 1.7) | 1.3 (-0.1, 2.7) |  | -0.5 (-1.6, 0.5) | -0.7 (-1.8, 0.5) |  | -0.3 (-1.4, 0.8) | -0.4 (-1.6, 0.8) |
| Notes: *k*, number of participants with data for at least one time point; *n*, number of observations from both time points. Higher score for each BASC outcome indicates more symptomatic behavior.  ^a^Models adjusted for maternal age at delivery, years in the U.S., education at baseline, marital status at baseline, depression at 9Y assessment; child age at time of assessment, poverty status at time of assessment, HOME score at 10.5Y assessment, agricultural applications of 11 pesticides included in prenatal assessment during the prenatal period. | | | | | | | | | | | | | | | |

| **Table S8.** Summary of associations of two-fold increase in pesticide use within 1 km of residence *during childhood* with maternal- and youth-reported behavioral and emotional problems at age 16 and 18 years using linear mixed effects Bayesian Hierarchical Modeling (BHM). | | | | | | | | | | | | |
| --- | --- | --- | --- | --- | --- | --- | --- | --- | --- | --- | --- | --- |
|  | Internalizing problems | | Depression | | Anxiety | | Externalizing problems | | Hyperactivity | | Attention problems | |
|  | Maternal | Youth | Maternal | Youth | Maternal | Youth | Maternal | Youth | Maternal | Youth | Maternal | Youth |
| Organophosphates |  |  |  |  |  |  |  |  |  |  |  |  |
| Acephate |  |  |  |  |  |  |  |  | **+** |  |  |  |
| Chlorpyrifos |  |  |  |  | **-** |  |  |  |  |  |  |  |
| Diazinon |  |  |  |  |  |  |  |  |  |  |  |  |
| Malathion |  |  |  |  |  |  |  |  |  |  |  | **-** |
| Oxydemeton methyl |  |  |  |  |  |  |  |  |  |  | **-** |  |
| Naled |  |  |  |  | **-** |  |  |  |  |  |  | **+** |
| Dimethoate |  | **+** |  |  |  |  |  |  |  | **+** |  |  |
| Carbamates |  |  |  |  |  |  |  |  |  |  |  |  |
| Methomyl |  |  |  |  |  |  |  |  |  |  |  |  |
| Pyrethroid |  |  |  |  |  |  |  |  |  |  |  |  |
| Permethrin |  |  |  |  |  |  |  |  |  |  |  |  |
| Neonicotinoid |  |  |  |  |  |  |  |  |  |  |  |  |
| Imidacloprid |  |  |  |  |  |  |  |  |  |  |  |  |
| Fungicide |  |  |  |  |  |  |  |  |  |  |  |  |
| Mn-Fungicides |  |  |  |  |  |  |  |  |  |  |  |  |
| Herbicide |  |  |  |  |  |  |  |  |  |  |  |  |
| Glyphosate | **+** | **+** |  | **+** | **+** | **+** |  |  |  |  |  |  |
| Notes: “+” indicates more symptomatic behavior; “-“ indicates less symptomatic behavior; blank cell indicates null association | | | | | | | | | | | | |

| **Table S9.** Adjusted^a^ associations [β (95% CI)] of two-fold increase in pesticide use within 1 km of residence *during pregnancy* with *maternal-reported* behavioral and emotional problems at age 16 and 18 years using linear mixed effects regression with all exposure variables included simultaneously. | | | | | | | | | | | | |
| --- | --- | --- | --- | --- | --- | --- | --- | --- | --- | --- | --- | --- |
|  |  | Internalizing problems^b^ |  | Depression^b^ |  | Anxiety^c^ |  | Externalizing problems^c^ |  | Hyperactivity^d^ |  | Attention problems^d^ |
| Organophosphates |  |  |  |  |  |  |  |  |  |  |  |  |
| Acephate |  | 0.1 (-1.4, 1.7) |  | -0.2 (-1.7, 1.4) |  | 0.6 (-1.0, 2.1) |  | -0.5 (-1.6, 0.7) |  | -0.3 (-1.5, 0.9) |  | -0.7 (-2.2, 0.7) |
| Chlorpyrifos |  | 0.7 (-0.6, 1.9) |  | 0.9 (-0.3, 2.2) |  | 0.5 (-0.7, 1.7) |  | 0.2 (-0.7, 1.1) |  | 0.2 (-0.7, 1.2) |  | -0.3 (-1.4, 0.9) |
| Diazinon |  | -0.1 (-1.5, 1.4) |  | -0.3 (-1.7, 1.2) |  | 0.4 (-0.9, 1.8) |  | 0.2 (-0.8, 1.3) |  | 0.0 (-1.1, 1.2) |  | 0.3 (-1.0, 1.6) |
| Malathion |  | 0.2 (-0.6, 1.0) |  | 0.6 (-0.3, 1.4) |  | -0.4 (-1.2, 0.4) |  | -0.1 (-0.7, 0.5) |  | -0.1 (-0.8, 0.5) |  | 0.1 (-0.7, 0.8) |
| Oxydemeton methyl |  | -0.1 (-2.4, 2.2) |  | 0.4 (-1.9, 2.8) |  | -0.4 (-2.7, 1.9) |  | 0.0 (-1.8, 1.7) |  | -0.4 (-2.3, 1.4) |  | 0.2 (-2.0, 2.4) |
| Dimethoate |  | 0.5 (-1.6, 2.5) |  | 0.2 (-1.9, 2.3) |  | 0.0 (-2.1, 2.0) |  | 0.4 (-1.2, 1.9) |  | 0.8 (-0.8, 2.4) |  | 1.3 (-0.6, 3.3) |
| Carbamates |  |  |  |  |  |  |  |  |  |  |  |  |
| Methomyl |  | 0.1 (-1.1, 1.3) |  | -0.1 (-1.3, 1.1) |  | 0.2 (-1.0, 1.4) |  | 0.5 (-0.4, 1.3) |  | 0.3 (-0.6, 1.3) |  | 0.0 (-1.1, 1.1) |
| Pyrethroid |  |  |  |  |  |  |  |  |  |  |  |  |
| Permethrin |  | -1.4 (-3.8, 1.1) |  | -1.2 (-3.7, 1.4) |  | -1.4 (-3.8, 1.0) |  | 0.3 (-1.5, 2.2) |  | 0.2 (-1.8, 2.1) |  | 0.5 (-1.8, 2.8) |
| Neonicotinoid |  |  |  |  |  |  |  |  |  |  |  |  |
| Imidacloprid |  | -0.3 (-3.3, 2.8) |  | 0.0 (-3.1, 3.1) |  | -1.1 (-4.1, 1.9) |  | -0.3 (-2.6, 1.9) |  | -0.7 (-3.1, 1.7) |  | -2.4 (-5.3, 0.5) |
| Fungicide |  |  |  |  |  |  |  |  |  |  |  |  |
| Mn-Fungicides |  | -0.1 (-1.4, 1.1) |  | -0.2 (-1.4, 1.1) |  | 0.0 (-1.2, 1.2) |  | -0.4 (-1.3, 0.6) |  | -0.1 (-1.1, 0.9) |  | 0.0 (-1.2, 1.1) |
| Herbicide |  |  |  |  |  |  |  |  |  |  |  |  |
| Glyphosate |  | 0.3 (-0.5, 1.0) |  | -0.1 (-0.8, 0.7) |  | 0.4 (-0.3, 1.1) |  | 0.4 (-0.2, 0.9) |  | 0.4 (-0.1, 1.0) |  | 0.4 (-0.2, 1.1) |
| Notes: *k*, number of participants with data for at least one time point; *n*, number of observations from both time points. Higher score for each BASC outcome indicates more symptomatic behavior.  ^a^Models adjusted for maternal age at delivery, years in the U.S., education at baseline, marital status at baseline, language of assessment, depression at 9Y assessment; child sex, child age at time of assessment, poverty status at time of assessment, HOME score at 10.5Y assessment.  *^b^n*=1,044; *k*=583  ^c^*n*=1,048; *k*=587  ^d^*n*=1,049; *k*=587 | | | | | | | | | | | | |

| **Table S10.** Adjusted^a^ associations [β (95% CI)] of two-fold increase in pesticide use within 1 km of residence *during pregnancy* with *youth-reported* behavioral and emotional problems at age 16 and 18 years using linear mixed effects regression with all exposure variables included simultaneously. | | | | | | | | | | |
| --- | --- | --- | --- | --- | --- | --- | --- | --- | --- | --- |
|  |  | Internalizing problems^b^ |  | Depression^c^ |  | Anxiety^c^ |  | Hyperactivity^d^ |  | Attention problems^d^ |
| Organophosphates |  |  |  |  |  |  |  |  |  |  |
| Acephate |  | 0.1 (-1.5, 1.8) |  | 0.3 (-1.4, 1.9) |  | -0.6 (-2.4, 1.1) |  | -0.1 (-1.5, 1.4) |  | 0.4 (-1.0, 1.9) |
| Chlorpyrifos |  | 1.1 (-0.2, 2.4) |  | 1.2 (-0.1, 2.4) |  | 0.6 (-0.8, 2.0) |  | -0.1 (-1.3, 1.0) |  | -0.1 (-1.2, 1.1) |
| Diazinon |  | 0.4 (-1.1, 1.9) |  | 0.3 (-1.1, 1.8) |  | 0.3 (-1.3, 1.9) |  | 0.0 (-1.3, 1.3) |  | 1.2 (-0.1, 2.5) |
| Malathion |  | 0.6 (-0.3, 1.4) |  | 0.5 (-0.3, 1.4) |  | 0.6 (-0.3, 1.5) |  | -0.1 (-0.9, 0.6) |  | 0.0 (-0.7, 0.8) |
| Oxydemeton methyl |  | -1.3 (-3.7, 1.2) |  | -0.9 (-3.4, 1.5) |  | -0.6 (-3.2, 2.0) |  | -0.7 (-2.9, 1.5) |  | -1.8 (-4.0, 0.4) |
| Dimethoate |  | -0.6 (-2.8, 1.6) |  | -1.2 (-3.4, 0.9) |  | -0.3 (-2.7, 2.0) |  | 0.7 (-1.2, 2.7) |  | 2.3 (0.3, 4.2) |
| Carbamates |  |  |  |  |  |  |  |  |  |  |
| Methomyl |  | 0.1 (-1.2, 1.3) |  | -0.5 (-1.8, 0.7) |  | -0.1 (-1.4, 1.3) |  | 0.4 (-0.7, 1.5) |  | -0.3 (-1.4, 0.8) |
| Pyrethroid |  |  |  |  |  |  |  |  |  |  |
| Permethrin |  | -0.5 (-3.1, 2.1) |  | -0.7 (-3.3, 1.8) |  | -1.2 (-4.0, 1.6) |  | -0.6 (-2.9, 1.6) |  | -1.9 (-4.2, 0.4) |
| Neonicotinoid |  |  |  |  |  |  |  |  |  |  |
| Imidacloprid |  | -0.3 (-3.5, 2.9) |  | 0.3 (-2.9, 3.4) |  | -0.8 (-4.2, 2.6) |  | 0.4 (-2.4, 3.3) |  | -1.6 (-4.5, 1.2) |
| Fungicide |  |  |  |  |  |  |  |  |  |  |
| Mn-Fungicides |  | 0.0 (-1.3, 1.3) |  | 0.2 (-1.1, 1.5) |  | 0.5 (-0.9, 1.9) |  | -0.2 (-1.3, 1.0) |  | 0.1 (-1.1, 1.2) |
| Herbicide |  |  |  |  |  |  |  |  |  |  |
| Glyphosate |  | 0.3 (-0.4, 1.1) |  | 0.4 (-0.4, 1.1) |  | 0.4 (-0.4, 1.3) |  | 0.4 (-0.2, 1.1) |  | 0.2 (-0.5, 0.8) |
| Notes: *k*, number of participants with data for at least one time point; *n*, number of observations from both time points. Higher score for each BASC outcome indicates more symptomatic behavior.  ^a^Models adjusted for maternal age at delivery, years in the U.S., education at baseline, marital status at baseline, depression at 9Y assessment; child sex, child age at time of assessment, poverty status at time of assessment, HOME score at 10.5Y assessment.  *^b^n*=1,027; *k*=584  ^c^*n*=1,031; *k*=584  ^d^*n*=1,030; *k*=584 | | | | | | | | | | |

| **Table S11.** Adjusted^a^ associations [β (95% CI)] of two-fold increase in pesticide use within 1 km of residence *during childhood (0-5 years)* with *maternal-reported* behavioral and emotional problems at age 16 and 18 years using linear mixed effects regression with all exposure variables included simultaneously. | | | | | | | | | | | | |
| --- | --- | --- | --- | --- | --- | --- | --- | --- | --- | --- | --- | --- |
|  |  | Internalizing problems^b^ |  | Depression^b^ |  | Anxiety^c^ |  | Externalizing problems^c^ |  | Hyperactivity^d^ |  | Attention problems^d^ |
| Organophosphates |  |  |  |  |  |  |  |  |  |  |  |  |
| Acephate |  | 1.1 (-0.9, 3.1) |  | 0.9 (-1.1, 2.9) |  | 1.2 (-0.8, 3.2) |  | 1.1 (-0.4, 2.6) |  | 2.2 (0.6, 3.8) |  | 1.0 (-0.8, 2.8) |
| Chlorpyrifos |  | -1.1 (-2.8, 0.7) |  | 0.0 (-1.7, 1.7) |  | -1.8 (-3.6, -0.1) |  | 0.2 (-1.1, 1.5) |  | 0.1 (-1.2, 1.5) |  | 1.1 (-0.5, 2.6) |
| Diazinon |  | 1.2 (-1.0, 3.4) |  | 1.4 (-0.8, 3.6) |  | 0.1 (-2.1, 2.3) |  | 0.0 (-1.7, 1.7) |  | 0.4 (-1.3, 2.1) |  | 1.4 (-0.6, 3.4) |
| Malathion |  | 0.3 (-0.8, 1.3) |  | 0.1 (-0.9, 1.2) |  | 0.8 (-0.2, 1.8) |  | -0.4 (-1.2, 0.4) |  | -0.6 (-1.4, 0.2) |  | -1.1 (-2.0, -0.1) |
| Oxydemeton methyl |  | 0.5 (-2.9, 3.8) |  | -0.4 (-3.7, 2.9) |  | 0.8 (-2.5, 4.1) |  | -1.3 (-3.8, 1.2) |  | -3.3 (-5.9, -0.6) |  | -2.5 (-5.5, 0.5) |
| Naled |  | -0.8 (-2.2, 0.5) |  | -0.2 (-1.5, 1.1) |  | -1.4 (-2.7, -0.1) |  | 0.5 (-0.5, 1.5) |  | 0.4 (-0.6, 1.4) |  | 1.0 (-0.2, 2.2) |
| Dimethoate |  | 1.1 (-1.4, 3.7) |  | -0.4 (-2.9, 2.1) |  | 1.3 (-1.2, 3.7) |  | 0.8 (-1.1, 2.7) |  | 1.0 (-1.0, 3.0) |  | -1.5 (-3.7, 0.8) |
| Carbamates |  |  |  |  |  |  |  |  |  |  |  |  |
| Methomyl |  | 0.2 (-1.5, 1.9) |  | 0.7 (-1.0, 2.4) |  | -0.6 (-2.3, 1.1) |  | -0.2 (-1.5, 1.1) |  | -0.5 (-1.8, 0.8) |  | -0.9 (-2.4, 0.7) |
| Pyrethroid |  |  |  |  |  |  |  |  |  |  |  |  |
| Permethrin |  | 0.3 (-2.2, 2.8) |  | -0.1 (-2.6, 2.4) |  | 0.7 (-1.8, 3.1) |  | -0.1 (-2.0, 1.8) |  | 1.0 (-1.0, 3.0) |  | -0.9 (-3.2, 1.3) |
| Neonicotinoid |  |  |  |  |  |  |  |  |  |  |  |  |
| Imidacloprid |  | 0.0 (-3.0, 2.9) |  | -0.3 (-3.2, 2.6) |  | 0.9 (-1.9, 3.7) |  | 0.3 (-1.8, 2.5) |  | 1.6 (-0.6, 3.9) |  | 1.9 (-0.7, 4.4) |
| Fungicide |  |  |  |  |  |  |  |  |  |  |  |  |
| Mn-Fungicides |  | -2.5 (-4.8, -0.3) |  | -1.6 (-3.9, 0.6) |  | -1.7 (-3.9, 0.5) |  | -0.6 (-2.3, 1.1) |  | 0.0 (-1.8, 1.7) |  | 0.4 (-1.6, 2.4) |
| Herbicide |  |  |  |  |  |  |  |  |  |  |  |  |
| Glyphosate |  | 1.4 (0.3, 2.4) |  | 0.6 (-0.5, 1.7) |  | 1.3 (0.3, 2.4) |  | 0.1 (-0.7, 0.9) |  | 0 (-0.8, 0.9) |  | -0.1 (-1.0, 0.9) |
| Notes: *k*, number of participants with data for at least one time point; *n*, number of observations from both time points. Higher score for each BASC outcome indicates more symptomatic behavior.  ^a^Models adjusted for maternal age at delivery, years in the U.S., education at baseline, marital status at baseline, language of assessment, depression at 9Y assessment; child sex, child age at time of assessment, poverty status at time of assessment, HOME score at 10.5Y assessment, agricultural applications of 11 pesticides included in prenatal assessment during the prenatal period.  ^b^*n*=793; *k*=427  ^c^*n*=796; *k*=427  ^d^*n*=797; *k*=427 | | | | | | | | | | | | |

| **Table S12.** Adjusted associations [β (95% CI)] of two-fold increase in pesticide use within 1 km of residence *during childhood (0-5 years)* with *youth-reported* behavioral and emotional problems at age 16 and 18 years using linear mixed effects regression with all exposure variables included simultaneously. | | | | | | | | | | |
| --- | --- | --- | --- | --- | --- | --- | --- | --- | --- | --- |
|  |  | Internalizing problems^b^ |  | Depression^c^ |  | Anxiety^d^ |  | Hyperactivity^e^ |  | Attention problems^e^ |
| Organophosphates |  |  |  |  |  |  |  |  |  |  |
| Acephate |  | 0.5 (-1.5, 2.5) |  | 0.2 (-1.7, 2.1) |  | -0.8 (-3.0, 1.4) |  | 1.2 (-0.7, 3.0) |  | -0.1 (-1.9, 1.8) |
| Chlorpyrifos |  | -1.2 (-2.9, 0.5) |  | -1.5 (-3.1, 0.1) |  | -1.1 (-2.9, 0.8) |  | 0.0 (-1.5, 1.6) |  | 0.1 (-1.4, 1.7) |
| Diazinon |  | 0.4 (-1.8, 2.6) |  | 0.5 (-1.6, 2.6) |  | 0.7 (-1.7, 3.1) |  | 0.4 (-1.6, 2.3) |  | -0.2 (-2.2, 1.8) |
| Malathion |  | -0.7 (-1.7, 0.3) |  | -0.3 (-1.3, 0.7) |  | -0.3 (-1.5, 0.8) |  | -0.4 (-1.3, 0.6) |  | -0.9 (-1.8, 0.1) |
| Oxydemeton methyl |  | -0.1 (-3.4, 3.2) |  | 0.6 (-2.5, 3.8) |  | 1.0 (-2.6, 4.6) |  | -2.3 (-5.3, 0.6) |  | -1.1 (-4.1, 1.9) |
| Naled |  | 0.5 (-0.8, 1.8) |  | 0.3 (-1.0, 1.5) |  | -0.4 (-1.8, 1.0) |  | 0.1 (-1.1, 1.3) |  | 1.4 (0.2, 2.6) |
| Dimethoate |  | 2.5 (0.0, 5.0) |  | 1.9 (-0.5, 4.3) |  | 1.9 (-0.8, 4.7) |  | 2.4 (0.2, 4.7) |  | 1.3 (-1.0, 3.6) |
| Carbamates |  |  |  |  |  |  |  |  |  |  |
| Methomyl |  | -1.4 (-3.1, 0.2) |  | -1.1 (-2.7, 0.5) |  | -0.2 (-2, 1.6) |  | -0.9 (-2.4, 0.6) |  | -1.0 (-2.6, 0.5) |
| Pyrethroid |  |  |  |  |  |  |  |  |  |  |
| Permethrin |  | 0.3 (-2.2, 2.8) |  | 0.1 (-2.3, 2.4) |  | 0.6 (-2.1, 3.3) |  | -0.3 (-2.5, 1.9) |  | 0.0 (-2.3, 2.3) |
| Neonicotinoid |  |  |  |  |  |  |  |  |  |  |
| Imidacloprid |  | -0.8 (-3.7, 2.1) |  | -1.6 (-4.3, 1.2) |  | -1.7 (-4.8, 1.4) |  | -0.2 (-2.8, 2.3) |  | 0.7 (-1.9, 3.4) |
| Fungicide |  |  |  |  |  |  |  |  |  |  |
| Mn-Fungicides |  | -0.3 (-2.6, 1.9) |  | -0.3 (-2.4, 1.8) |  | -0.2 (-2.6, 2.2) |  | 0.6 (-1.5, 2.6) |  | 0.5 (-1.6, 2.5) |
| Herbicide |  |  |  |  |  |  |  |  |  |  |
| Glyphosate |  | 1.0 (-0.1, 2.0) |  | 1.3 (0.3, 2.3) |  | 0.9 (-0.3, 2.1) |  | -0.6 (-1.6, 0.4) |  | -0.1 (-1.1, 0.9) |
| Notes: *k*, number of participants with data for at least one time point; *n*, number of observations from both time points. Higher score for each BASC outcome indicates more symptomatic behavior.  ^a^Models adjusted for maternal age at delivery, years in the U.S., education at baseline, marital status at baseline, depression at 9Y assessment; child sex, child age at time of assessment, poverty status at time of assessment, HOME score at 10.5Y assessment, agricultural applications of 11 pesticides included in prenatal assessment during the prenatal period.  ^b^*n*=783; *k*=426  ^c^*n*=786; *k*=426  ^d^*n*=785; *k*=426  ^e^*n*=784; *k*=426 | | | | | | | | | | |

**Figure S1.** Spearman correlation coefficients of agricultural pesticide use within 1 km of home during the prenatal period.

**Figure S2.** Spearman correlation coefficients of agricultural pesticide use within 1 km of home during the postnatal period.
